# Modular stayed 3D-lattice structures manufactured by MEX-AM: buckling behaviour and energy absorption

**DOI:** 10.1038/s41598-026-53719-6

**Published:** 2026-06-16

**Authors:** Yating Ou, Anas Yousif, Till Haroske, Milan Thiele, Narges Panjalipoursangari, Christina Völlmecke

**Affiliations:** 1 Stability and Failure of Functionally Optimized Structures Group (SVFS Group), Technische Universität Berlin, Institut für Mechanik, Einsteinufer 5, 10587 Berlin, Germany; 2 Continuum Mechanics and Materials Theory Group, Technical University of Berlin, Institut für Mechanik, Einsteinufer 5, 10587 Berlin, Germany; 3 Technische Universität Berlin, Institut für Mechanik, Einsteinufer 5, 10587 Berlin, Germany

**Keywords:** Lattice structures, Modular design, Design for recycling, Buckling behaviour, Energy absorption, Engineering, Materials science

## Abstract

Slender lattice structures offer significant advantages in mechanical applications due to their high stiffness and energy absorption capabilities; however, they are inherently prone to buckling. Although stay-based reinforcement concept have proven effective in two-dimensional lattice, extending them to three-dimensional (3D) structures presents challenges in both design and manufacturing. This study aims to develop a material-extrusion manufactured stayed 3D-lattice based on a modular design that enables non-planar stay arrangements. The goal is to improve the ultimate load capacity and energy-absorption performance of the lattice through the introduction of stays. Material-extrusion additive manufacturing was used to fabricate modular stayed lattice specimens, followed by uniaxial compression testing and digital image correlation to evaluate mechanical performance and deformation behaviour. Three types of connectors and three types of stayed unit cells were fabricated through a modular strategy and printing parameter optimisation, and assembled into six distinct lattice configurations. Buckling behaviour, relative density, relative strength, and energy absorption were studied. The results demonstrate that the stay concept can be effectively applied to 3D-lattices, increasing the ultimate load by up to a factor of 3.53 and enhancing energy-absorption capability to approximately 81%. The buckling behaviour could be tuned across five lattice configurations by varying unit cell types and additional reinforcement strategies. The connectors remained intact without observable micro-cracking, which is favourable for recyclability. Overall, the proposed modular stayed 3D-lattice framework offers a recyclable and effective strategy to enhance stability and energy absorption, providing fundamental data for selecting optimal configurations for intended applications.

## Introduction

Lightweight structures are essential in modern engineering for achieving high strength and stiffness with minimal material use^1–3^. Among them, slender lattice structures offer excellent strength-to-weight efficiency and tunable mechanical properties, achieved by repeating unit cells arranged in a regular pattern^4,5^. There are many types of lattice structures that can be classified based on their architecture and mechanical behaviour. Architecturally, lattices may be beam/strut-based, sheet-based, or surface-based, each offering distinct geometric characteristics and manufacturing possibilities^6–9^. Mechanically, they can be stretch-dominated, bending-dominated, or mixed, depending on how loads are transferred through the structure^10^. These modifications allow lattice designs to be tailored for specific performance goals such as high stiffness and strength^11^, energy absorption^12^, and auxetic behaviour^13^. With these unique mechanical properties, lattice structures have found extensive applications across diverse fields such as biomedical engineering^14^, the automotive industry^15,16^, and aerospace and aviation^17^. The rise of 3D-printing has made it possible to fabricate complex lattice geometries that were previously unachievable by traditional methods^18^.

**Fig. 1:**
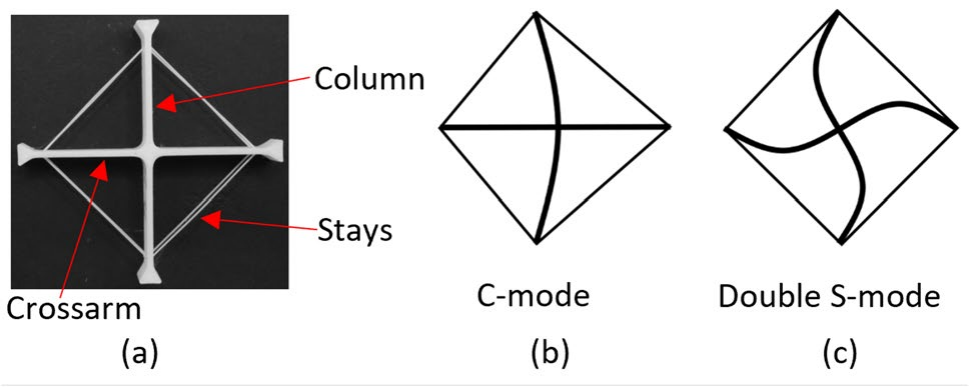
Illustration of the initial configuration and buckling responses of stayed unit cells: (**a**) 3D-printed UC in undeformed configuration, (**b**) C-mode induced by weak stay restraint and (**c**) double-S mode induced by strong stay restraint.

A novel strut-based, bending-dominated lattice structure was presented by Ou et al.^19^, featuring a lightweight, high-performance design composed of unit cells made of stayed slender columns. As shown in Fig. 1a, the stayed unit cell consist the column, crossarm and the diagonal stays. The buckling behaviour of this structure is primarily determined by the function and effectiveness of the stays within the structure. With weak stay restraint, the unit cells buckle in a C-mode (Fig. 1b) under low loads. As the restraint strengthens beyond a threshold, the deformation shifts to a double-S mode (Fig. 1c), enabling a much higher compressive capacity.

The single unit cells and the corresponding 2D-lattice structures have been investigated in previous studies^19,20^. The material-extrusion additive manufacturing (MEX-AM) method enables the printing of continuous diagonal stays in mid-air without support, using the bridging technique. The stayed lattice structure offers clear mechanical advantages, notably increasing the ultimate load capacity with only a minor addition of material mass (by adding the diagonal stays). It also demonstrates excellent energy absorption capability under compressive loading, making it highly efficient for lightweight structural applications^20^.

Given these promising mechanical advantages, it is worthwhile to further develop this concept into a full three-dimensional (3D) lattice structure, which could not only broaden its potential engineering applications but also enhance its structural efficiency and functional adaptability in complex loading environments. Compared to 2D-lattice structures, 3D-lattice structures offer better multidirectional load distribution^21,22^, improved buckling stability due to out-of-plane support^23,24^, and higher energy absorption capacity resulting from a more gradual deformation mechanism^25,26^.

However, when extended to a 3D-lattice structure, printing it in a single build becomes highly challenging. The stays cannot be printed continuously in non-planar directions because conventional slicers generate planar layers. As a result, stays that are not aligned with the layer plane are divided into very short segments, leading to discontinuous extrusion paths that weaken their load-bearing capacity^27^.

The aim of this study is to develop and investigate a fully three-dimensional stayed lattice structure based on the previously studied 2D configurations. The proposed solution adopts a modular approach, allowing the 3D-lattice to be constructed from repeatable and easily fabricated units. The modular design concept has been widely recognized in lattice and metamaterial research for improving manufacturability, scalability, and repairability^28,29^. Modular design enables the selective replacement of damaged or end-of-life components^30,31^, while separation by material type facilitates efficient recycling, making this approach highly suitable for sustainable additive manufacturing^32^.

This study has two main objectives:

1. To design a 3D stayed lattice structure using a modular approach with material-extrusion additive manufacturing (MEX).
2. To experimentally investigate its buckling behaviour and evaluate the effectiveness of the modular design.

**Fig. 2:**
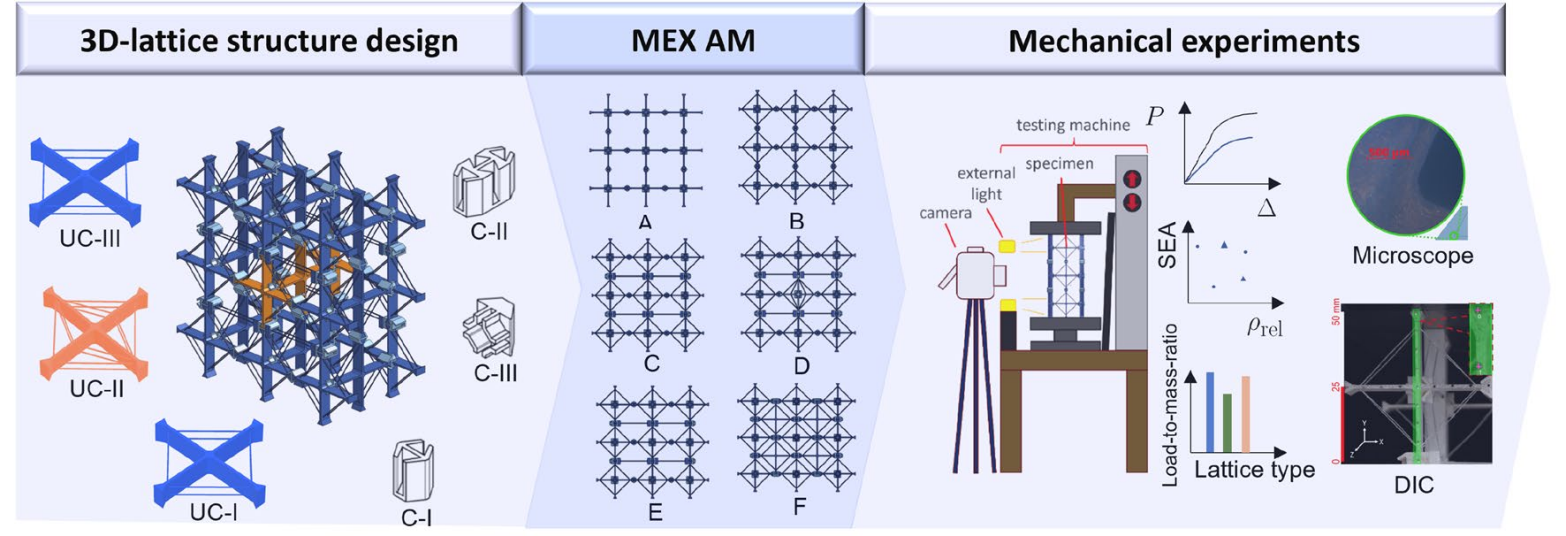
Overall workflow of the study: modular lattice design with different connector and unit cell types, fabrication via MEX-based additive manufacturing using PLA, and subsequent uniaxial compression testing assisted by DIC and microscopic analysis.

The workflow of this study is illustrated in Fig. 2. First, a modular design approach was employed for the three-dimensional lattice using three types of connectors (enabling connections in different orientations) and three unit cells (regulating local buckling behavior). Subsequently, the 3D computer-aided design (CAD) models were fabricated using polylactic acid (PLA) material via the material-extrusion additive manufacturing (MEX-AM) process, with carefully optimized printing parameters to ensure high printing quality. Finally, uniaxial compression tests were conducted on six different configurations to identify the buckling and post-buckling behaviour of the structures, assisted by digital image correlation (DIC) and microscopic observation.

## Methodology

This section outlines the methodology employed for designing and evaluating three-dimensional lattice structures. The methodology encompasses two parts: 3D-lattice structure design and mechanical experiments.

### 3D-lattice structure design

This section outlines the design of the 3D lattice structure, including the development and modelling of individual components, the structural assembly of the lattice system, and the definition of the test series.

All components were fabricated using material-extrusion additive manufacturing, encompassing filament-based techniques such as fused filament fabrication (FFF). The 3D-models were designed in Onshape^33^, which facilitated collaborative work by enabling multiple contributors to design simultaneously. The model was sliced with the open-source slicer, PrusaSlicer version 2.9, to generate the G-code required for 3D-printing. They were printed using a Prusa i3 MK3S+ 3D printer (Prusa Research a.s., Prague, Czech Republic) with Silver Polylactic Acid (PLA) filament (Prusa Research).

#### Component design

The design of the 3D-lattice structure components is introduced, including the unit cells, connectors and bracing. The underlying design concept and the corresponding improvements are systematically discussed.

##### Stayed unit cells

The design concept of the stayed unit cells is based on the previous work of Ou et al.^19^, and has been modified to meet the requirements for assembling the 3D-lattice structure. To successfully print the stays of the unit cell, it requires the printer to perform so-called bridging, in which filament is printed in the air, without any support structure. They also are printed under low velocity, as a single continuous shape with as little contact area to the columns and crossarms as possible in order to avoid them sticking together.

**Fig. 3:**
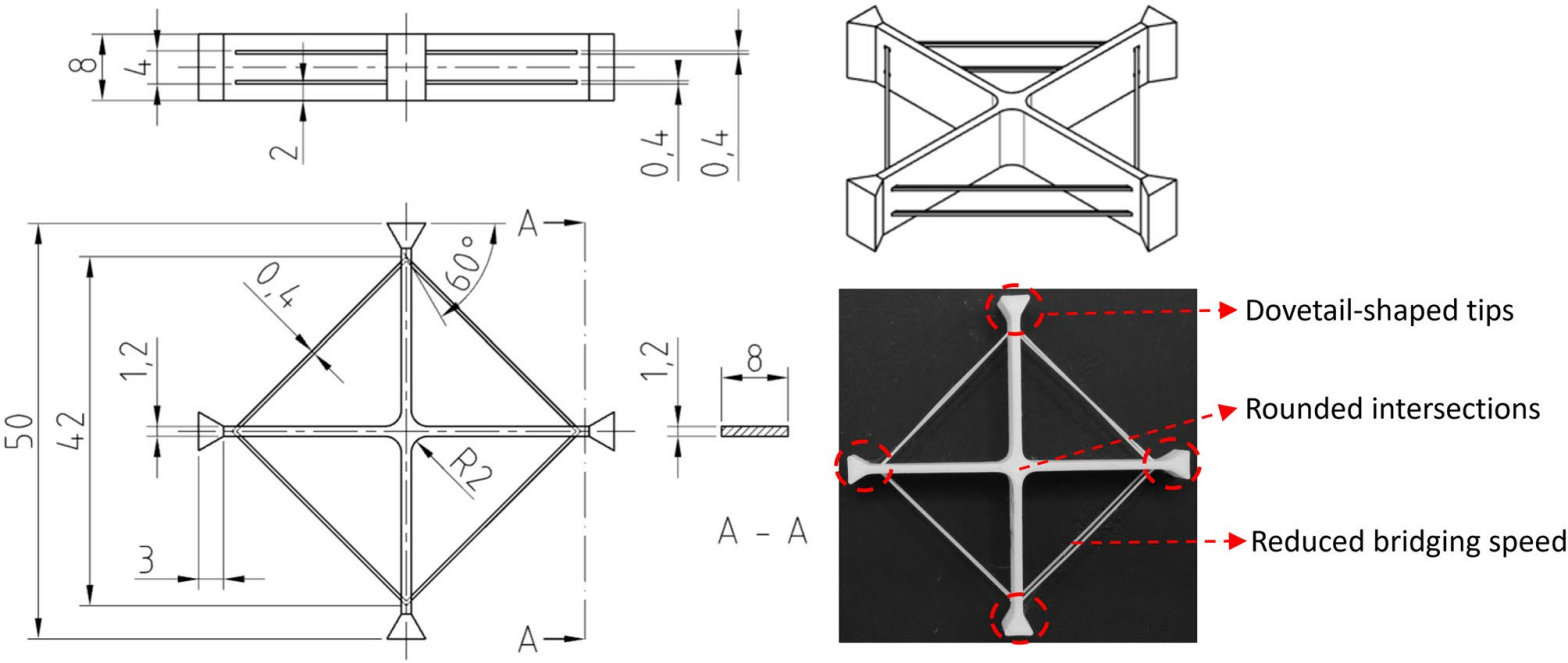
Technical drawing of the standard unit cell (UC) and the corresponding printed object; all dimensions in mm.

The technical drawing of the new designed stayed unit cells is presented in the Fig. 3, and has been modified in the following three aspect:

- *Dovetail-shaped tips*
  Dovetail-shaped tips simplify the assembly process and enable the easy design of connectors, offering greater structural compatibility than rounded end points.
- *Rounded intersections*
  The rounded intersection has been applied in this structure based on the previous study by Ou et al.^20^, who demonstrated that rounded intersections perform better under high-stress conditions compared with right-angled intersections. The rounded geometry helps reduce stress concentration at sharp corners and prevents sudden fracture under high compressive loads, a failure mode that can occur in 3D-lattice structures.
- *3D-printing parameter settings*
  Based on the printing parameters reported by Ou et al.^19^, the slicer settings in this study were iteratively adjusted during pilot trials to achieve consistent, high-quality print results. The final parameters are summarized in Table 1. The new approach introduces differentiated printing speeds for the various unit cell components. Compared with the previous work^19^, the bridging speed (*i.e.*, the printing speed of the stays) was reduced, while the printing speeds of the columns and crossarms were increased.

**Table 1:** Overview of PrusaSlicer printing settings.

Settings	Value
Layer height	0.2 mm*
First layer height	0.2 mm*
Perimeters	2
Fill density	30 %
Fill pattern	Rectilinear
First layer density	90 %
Perimeters speed	$80\ \textrm{mm}/\textrm{s}^2$
Small perimeters speed	$40\ \textrm{mm}/\textrm{s}^2$
Bridging speed	10 mm/s
First layer speed	40 mm/s
Acceleration perimeters	$800\ \textrm{mm}/\textrm{s}^2$
Acceleration bridge	$100\ \textrm{mm}/\textrm{s}^2$
Acceleration first layer	$1000\ \textrm{mm}/\textrm{s}^2$
Extrusion width default	0.45 mm
Extrusion width first layer	0.45 mm
Extrusion width infill	0.45 mm
Extrusion width top solid infill	0.45 mm
Nozzle temperature first layer	200 $^\circ$C
Nozzle temperature other layers	200 $^\circ$C
Bed temperature fist layer	55 $^\circ$C
Bed temperature other layers	55 $^\circ$C

* 0.15 mm for the connectors.

**Fig. 4:**
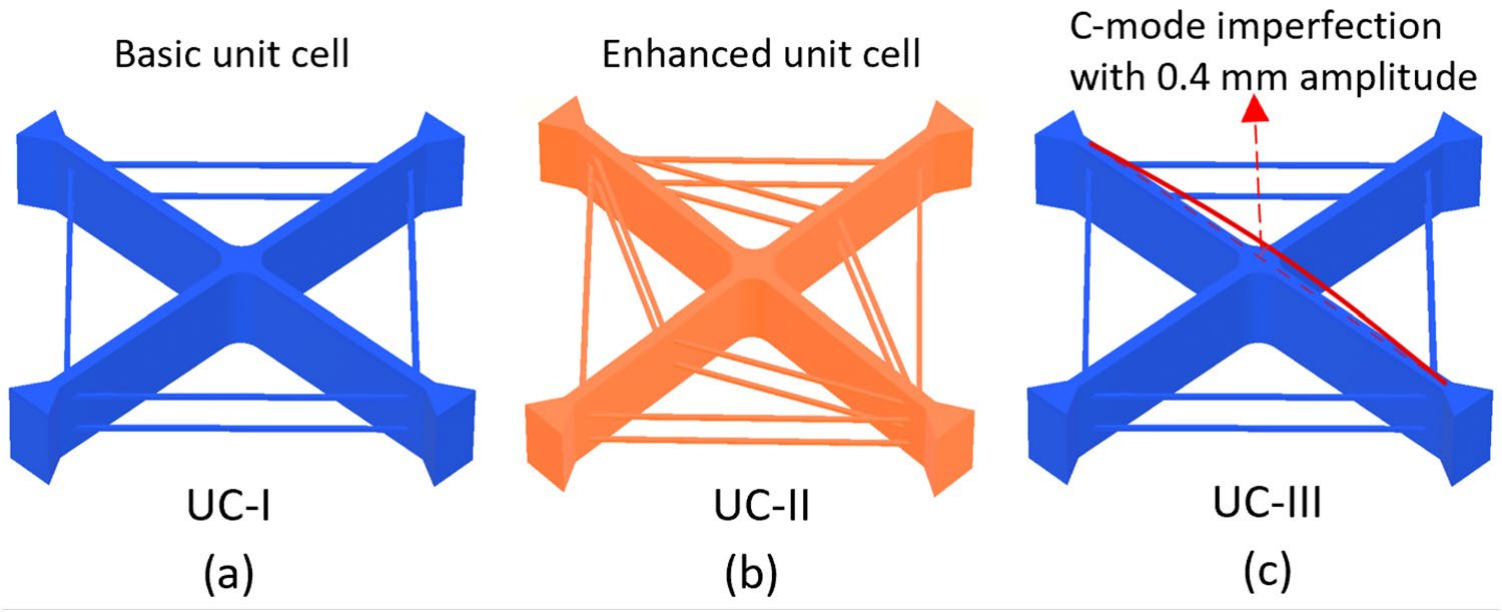
Three types of unit cells: (**a**) UC-I: basic unit cell^19^, (**b**) UC-II: enhanced unit cell with additional stays^34^ and (**c**) UC-III: imperfect unit cell with C-mode imperfection^20^.

Three different unit cell (UC) types are used in this study, and their corresponding technical drawings are shown in Fig. 4. The basic UCs (see Fig. 4a) represent the most commonly used configuration and serve as the primary building blocks of the 3D lattice. Enhanced and imperfect UCs (see Fig. 4b,c) are introduced only in the central region of the lattice to investigate their effect on tuning the local buckling behaviour of the 3D structure. Table 2 summaries the three types of unit cells used in this study, along with their corresponding weights and printing times.

##### Connector

**Fig. 5:**
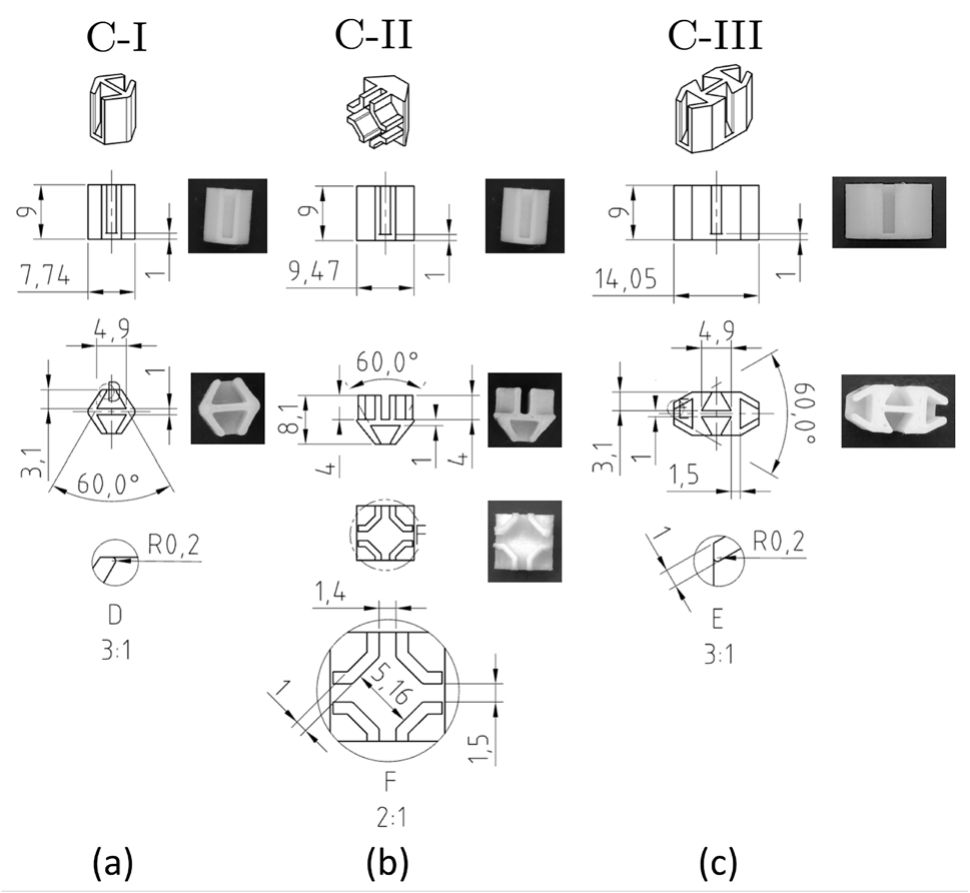
Technical drawings of the three different connectors and the corresponding printed objects: (**a**) C-I: lateral connector, (**b**) C-II: vertical connector and (**c**) C-III: four-way connector; all dimensions in mm.

To connect multiple stayed unit cells in different directions, three types of connectors were designed based on the dovetail connection principle, as shown in Fig. 5, where the technical drawings and corresponding printed components are presented. For this purpose, every unit cell (UC) has respective dovetail-shaped counterparts, at the tips of every column and crossarm.

**Fig. 6:**
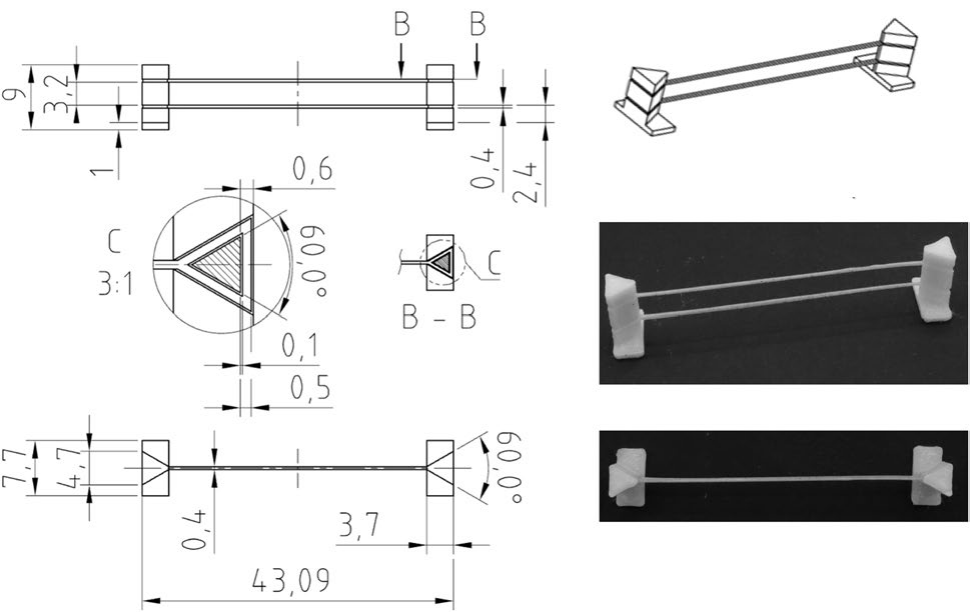
Technical drawing of the bracing and the corresponding printed object; all dimensions in mm.

- *C-I: lateral connector*
  For lateral directions, a lateral connector (C-I) with two opposing dovetail slots was developed, as shown in Fig. 5a. The structure can be assembled by sliding the parts into each other.
- *C-II: vertical connector*
  To create a lattice structure in vertical direction, a vertical connector (C-II, see Fig. 5b) with a cross and a dovetail slot on top was developed as well. It connects the tip of one UC with the cross in the middle of another UC.
- *C-III: 4-way connector*
  To stiffen the structure, a new connector type was developed for a number of pilot studies, as seen in Fig. 5c. It can be used primarily as a lateral connector, but also includes a bracing (see Fig. 6) between two stayed unit cells serving as a reinforcement, similarly to the stays working on a local scale within the cells. This connector is called a 4-way-connector.

Table 2 summaries the three connector types used in the 3D-lattice design, including their functions, weights, and printing durations.

**Table 2:** Overview of unit cells and connector types with corresponding weight and printing time.

Type	Description	Weight [g]	Printing time [s]
UC-I	Basic UCs	1.27 (scale), 1.47 (slicer)	780
UC-II	Enhanced UCs	1.3 (scale), 1.5 (slicer)	780
UC-III	Imperfect UCs	1.31 (scale), 1.5 (slicer)	780
C-I	Lateral connector	0.25 (scale), 0.35 (slicer)	300
C-II	Vertical connector	0.29 (scale), 0.58 (slicer)	600
C-III	4-way connector	0.54 (scale), 0.67 (slicer)	480

##### Bracing

The bracing elements serve as horizontal reinforcement between adjacent unit cells (UCs) and are designed to enable control of the buckling direction along different columns within the 3D lattice. The technical drawing of the bracing component is shown in Fig. 6. The bracing is connected to the main structure through a four-way connector C-III, linking it to the corresponding neighbouring elements.

#### Assembly

**Fig. 7:**
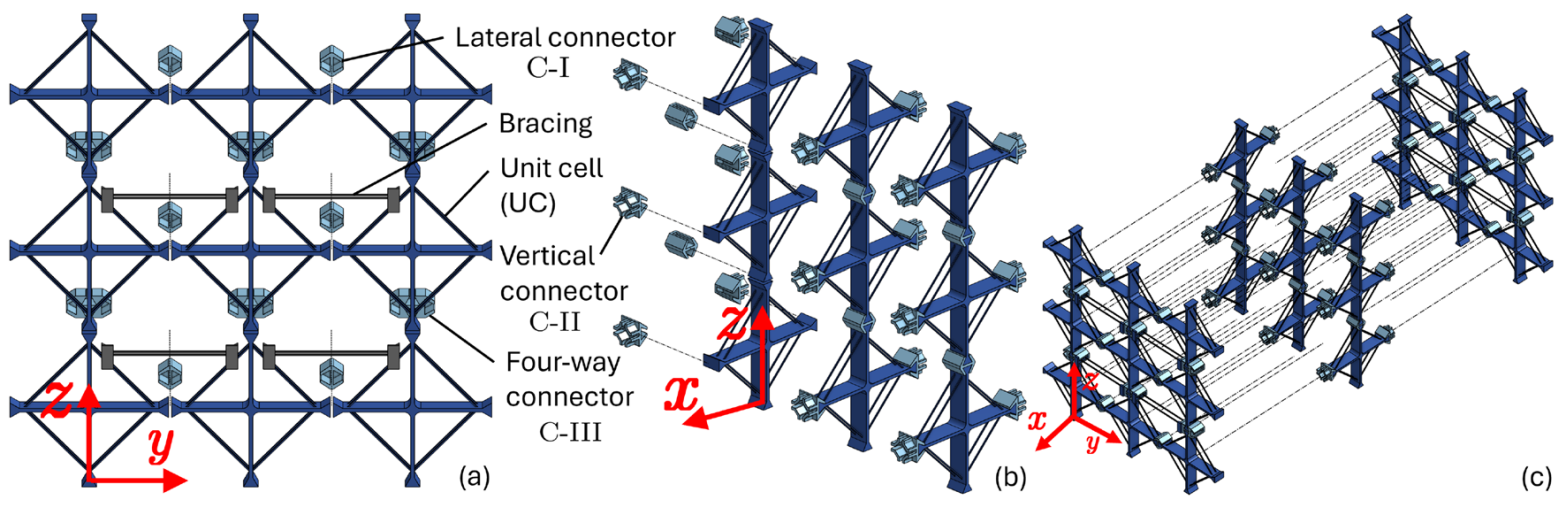
Assembly steps of 3D-lattice structure.

As illustrated in Fig. 7, the overall assembly procedure of the three-dimensional lattice is summarised below:

- Step 1 (Fig. 7a): the fabrication begins with two planar 3 $\times$ 3 lattice grids, which serve as the front and rear faces of the structure. Each grid is composed of repeating unit cells (UCs) that define the fundamental geometrical module of the lattice. In the *y*-direction, the lateral connector C-I is used, whereas in the *x*-direction the four-way connector C-III is employed. In special cases, the bracing elements (highlighted in gray) or additional unit cells (UCs) can also be connected to the four-way connector.
- Step 2 (Fig. 7b):In the second step, three vertical columns are assembled, each consisting of three basic unit cells (UCs). In the *z*-direction, the lateral connector C-II combines these three unit cells into a single column. In the *x*-direction, the vertical connector C-II is used, enabling the planar 3 $\times$ 3 lattice grids from (a) to be connected to the column form (b).
- Step 3 (Fig. 7c): finally, the two planar 3 $\times$ 3 grids from (a) are joined with the three vertical connector columns from (b). This step interlinks all components along the *x*–, *y*–, and *z*–axes, resulting in a fully three-dimensional lattice architecture.

The modular connector system ensures precise alignment and enables detachable assembly of the structure without additional fasteners or adhesives.

#### Test series design

**Fig. 8:**
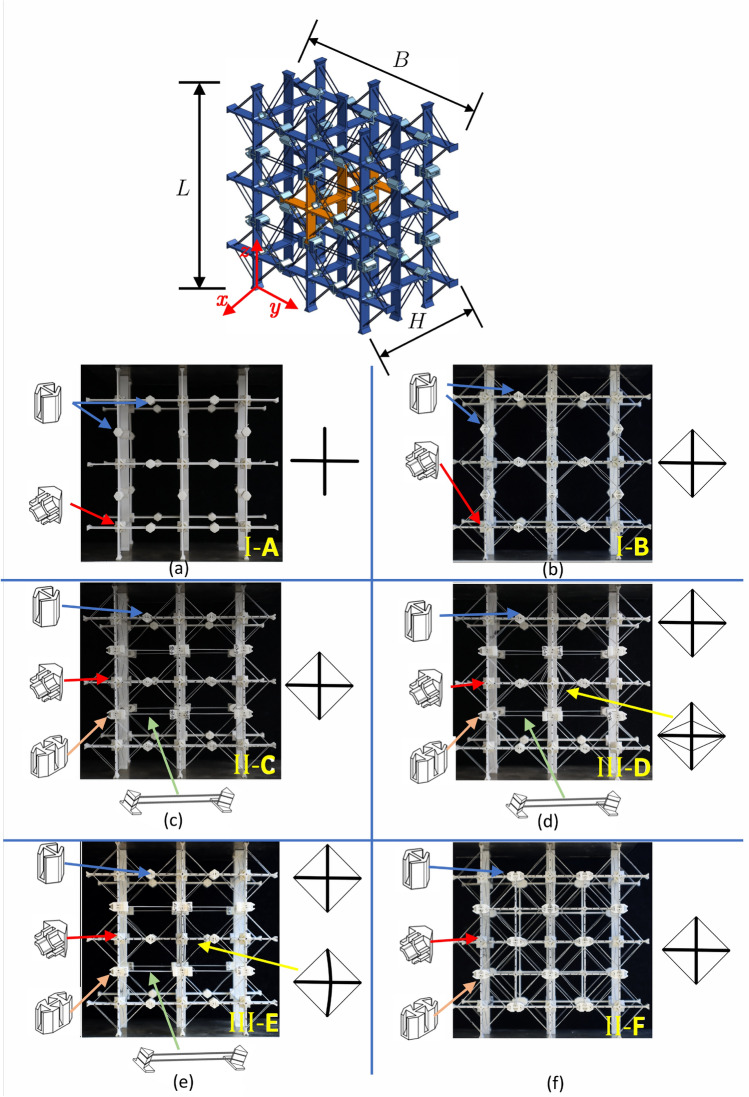
Six types of lattice structures: (**a**) Type I-A: basic 3D-lattice without stays, used as a reference configuration, (**b**) Type I-B: stayed 3D-lattice, verifying the effectiveness of the stayed concept, (**c**) Type II-C: standard stayed lattice with additional horizontal bracings, (**d**) Type III-D: lattice with reinforced unit cells at the center, (**e**) Type III-E: lattice with imperfect stayed unit cells at the center and (**f**) Type II-F: standard stayed lattice with additional basic unit cells.

A total of six types of lattice structures were investigated in this study, as shown in the Fig. 8. According to their different design objectives, they can be classified into three groups:

- *Group I: basic stayed 3D-lattice*:
  This group aims to verify whether the stayed concept functions effectively in a three-dimensional configuration. A lattice without stays (Type I-A, Fig. 8a) and a lattice with stays (Type I-B, Fig. 8b) using the standard stayed unit cells were compared to evaluate the feasibility of the assembly concept. Type I-B has been tested three times with repetition to ensure the reliability of the results.
- *Group II: 3D-lattices with additional components*:
  In this group, supplementary structural elements were introduced as a means to further enhance the mechanical performance. The Type II-C in Fig. 8c employed standard stayed unit cells, with additional horizontal bracings introduced via the four-way connectors linking adjacent cells. The Type II-F in Fig. 8f was also based on the standard stayed unit cells, but incorporated extra basic unit cells connected through the four-way connectors to further reinforce the structure.
- *Group III: 3D-lattices with different unit cells*:
  This group focuses on tuning the configuration of the unit cells to study the influence of structural variation on overall behaviour. Type III-D in Fig. 8d can be distinguished by the inclusion of three reinforced unit cells with additional stays positioned at the center of the lattice structure. This reinforced unit cells has been evidenced in the previews work, the load-bearing capacity can be increasing by 70.11% compared with the standard unit cell configuration^34^. Type III-E in Fig. 8e employed imperfect stayed unit cells, in which the columns were intentionally fabricated with C-mode imperfections. Previous studies have shown that this specific C-mode imperfection can also enhance the ultimate load capacity of the structure^20^. The incorporation of enhanced unit cells allows for the tuning of the buckling behaviour of the 3D-lattice structure

An overview of the six investigated lattice structure types, including the numbers of different unit cells, connectors, and bracings, is provided in Table 3. Descriptions of each component can be found in Table 2. Together with the assembly steps shown in Fig. 7, the configuration of each lattice structure is clearly illustrated.

**Table 3:** Overview of the six lattice structure configurations, including component counts and total weight.

Type	Number of UCs			Number of connectors			Bracings	Weight (g)
	UC-I	UC-II	UC-III	C-I	C-II	C-III		
I-A	27*	–	–	30	18	–	–	44.83
I-B	27	–	–	30	18	–	–	46.21
II-C	27	–	–	18	18	12	8	54.14
III-D	24	3	–	18	18	12	8	54.19
III-E	24	–	3	6	18	24	8	54.23
II-F	35	–	–	6	18	24	-	63.40

*The unit cells in Type I-A are without stays.

**Fig. 9:**
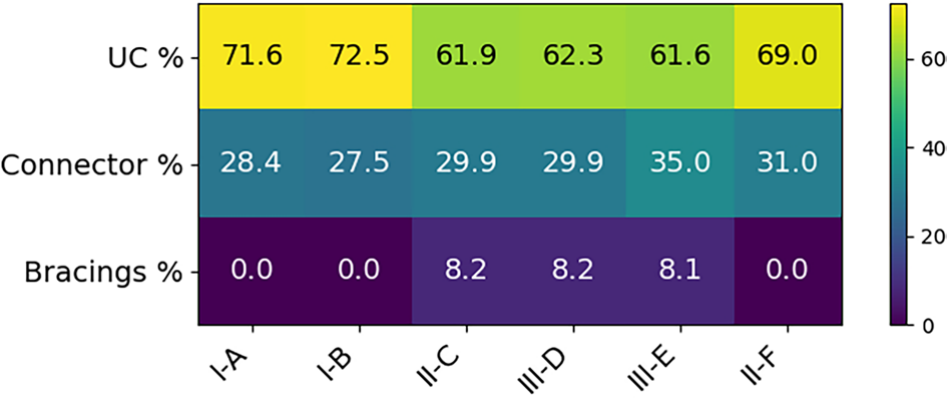
Heatmap of component mass distribution for different lattice configurations, showing the percentage contributions of UCs, connectors, and bracings across all types.

Figure 9 illustrating the relative mass distribution of structural components for different lattice configurations, including unit cells (UCs), connectors, and bracings.

### Uniaxial compression tests

The uniaxial compression tests, including the testing setup and data analysis, were conducted to evaluate the buckling behaviour of the 3D-lattice structures.

#### Test setting with DIC

**Fig. 10:**
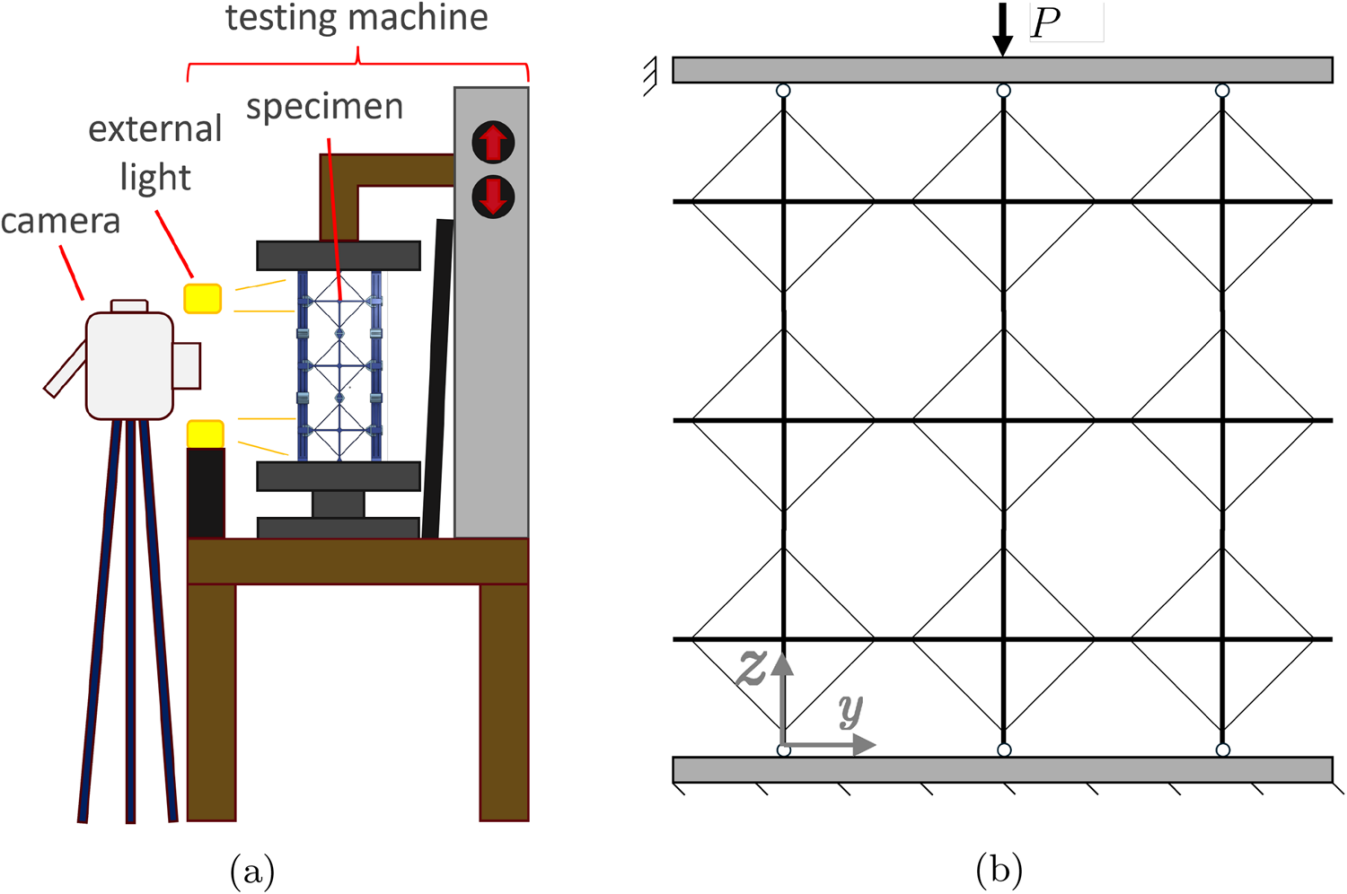
Test setting for uniaxial compression test: (**a**) schematic illustration of the test setup and (**b**) mechanical sketch of the specimen illustrating the boundary conditions.

Quasi-static uniaxial compression tests were conducted to investigate the buckling behaviour of stayed lattice structures with different configurations. The tests were performed using a ZwickRoell Z2.5 mechanical testing machine (ZwickRoell GmbH & Co. KG, August-Nagel-Straße 11, 89079 Ulm, Germany). All test series were carried out under displacement-controlled loading with an end-shortening rate of 0.2 mm/min. This low loading rate allows for a clear observation of the buckling behaviour. The boundary conditions of the test are illustrated in Fig. 10b, and the schematic setup of the experimental arrangement is shown in Fig. 10a.

The related test setting for the compression test is summarized in the Table 4.

**Table 4:** Uniaxial compression test settings.

Settings	Value
Total compressive travel	7 mm
Compression speed	0.2 mm/min
Camera Interval	0.02 mm/s

In order to observe the deformation changes and quantify the displacement magnitude during the buckling and post-buckling phases, digital image correlation (DIC) was applied in the experiments. As shown in Fig. 10a, a digital camera mounted on a tripod was connected to the testing machine, automatically capturing images at specified time intervals. These images record the deformation evolution throughout the experiment, enabling detailed analysis of the structural response during loading.

**Fig. 11:**
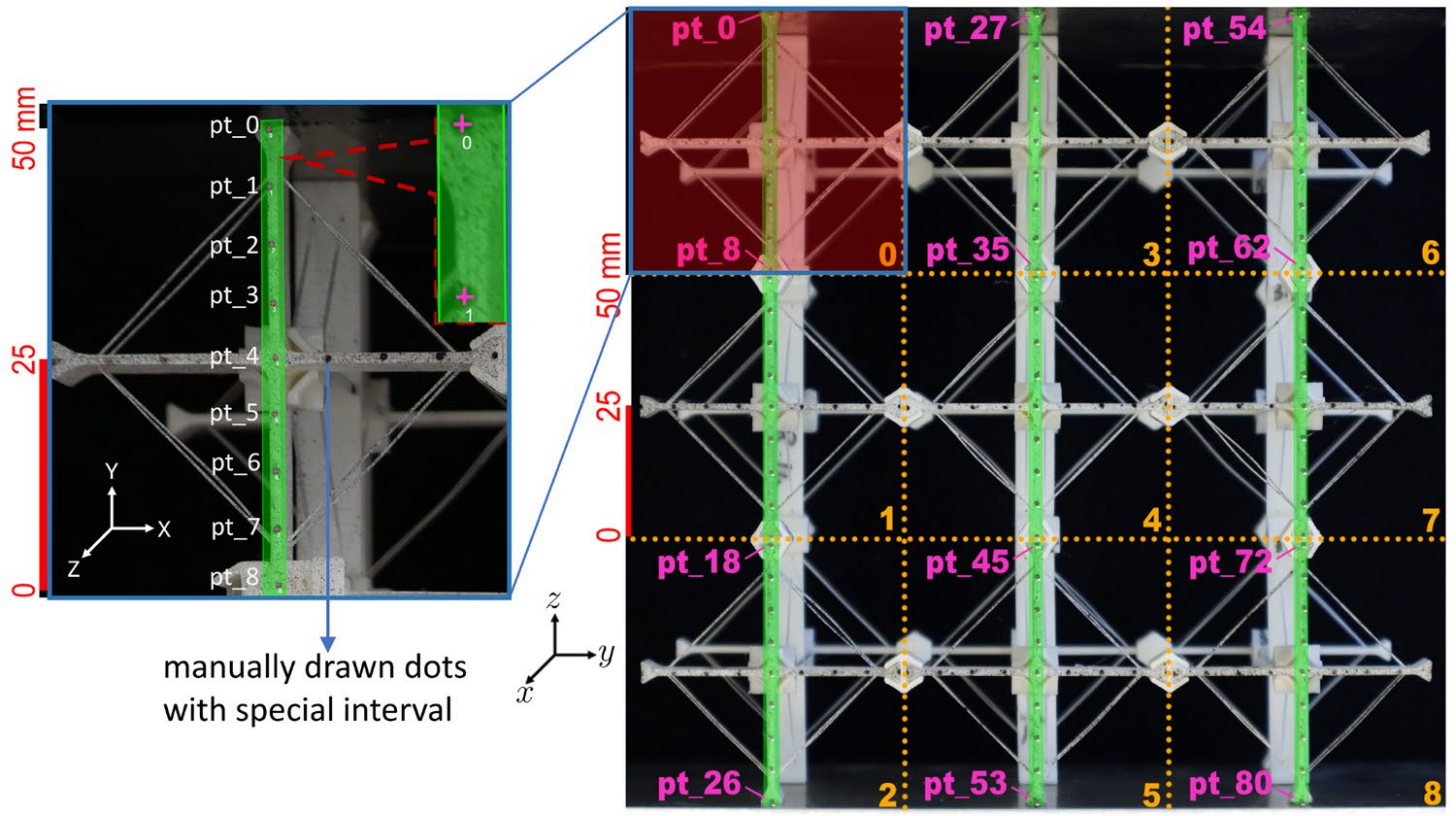
Speckle system for DIC: subdivision of the lattice structure and numbering of the manually applied speckles.

In order to track the deformation, the specimen was preprocessed by applying a random speckle pattern using spray paint and manually drawn dots^34,35^. As illustrated in Fig. 11, the structure was divided into nine regions ($0 - 8$) to allow a localized deformation analysis and ensure a clear display of the results. Each column within a unit cell is marked with nine equally spaced tracking points (pt_0 to pt_8) Therefore, the complete 3 $\times$ 3 lattice structure consists of 81 measurement points and three columns. As displayed in Fig. 11, each tracking point was marked as a plot point (pink cross) in the digital image correlation Engine (DICe 2.0) software^36^. The three columns were defined as regions of interest (ROI) and marked in green. Images of the format JPEG were used to process the data.

The settings used in DICe 2.0 are listed in the following Table 5:

**Table 5:** Parameter settings used for the DIC analysis in DICe 2.0^36^.

Settings	Value
Analysis mode	Subset-based full-field
Initialization method	Feature matching
sssig threshold	40
Subset size	19 (pixels)
Step size	9 (pixels)
Shape function	Translation (enabled)
Strain	Compute strain (enabled), gauge size: 30 (pixels)
Filtering	Gauss filter images (enabled), filter window: 5 (pixels)

After the experiments, a microscope (Olympus EX51 microscope, fitted with an AxioCam MRc camera by Carl Zeiss, Germany) was used to examine the manufacturing quality and analyses the failure surface of the specimens.

#### Data analysis

The data analysis comprises both the quantitative evaluation of various mechanical properties obtained from compression tests and the quantitative assessment of deformation behaviour through digital image correlation (DIC), enabling detailed observation and measurement of buckling characteristics.

The results of the compression tests for different 3D-lattice configurations were plotted as the relationship between the load *P* and the end-shortening $\Delta$ to illustrate the buckling and post-buckling behaviour.

The load-to-mass ratio expresses the relationship between the maximum load-bearing capacity ($P_{\textrm{max}}$) and the mass (*m*) of the structure. It serves as a dimensionless indicator of structural efficiency, showing how much load a structure can sustain relative to its own mass. A higher ratio indicates a lighter yet stronger design.

The relative density of the 3D-lattice, defined as the ratio of the lattice mass, $m_{\textrm{lattice}}$, to the mass of a fully solid structure, $m_{\textrm{solid}}$, of the same external dimensions^20,37^:

1 $$\begin{aligned} \rho _{\textrm{rel}} = \frac{m_{\textrm{lattice}}}{m_{\textrm{solid}}}=\frac{m_{\textrm{lattice}}}{BLH\rho _{\textrm{solid}}}\,, \end{aligned}$$

where the mass of the lattice structure was obtained from the measured data and is summarized in Table 3. The geometric dimensions of the 3D-lattice *B*, *L* and *H* are illustrated in the Fig. 8. The solid density of the PLA material $\rho _{\textrm{solid}}$ is 1.24 g/cm$^{3}$.

The relative strength of the 3D-lattice is defined as the ratio of the stress of the lattice structure $\sigma _{\textrm{lattice}}$ to that of a fully solid specimen with the same external dimensions $\sigma _{\textrm{solid}}$. The stress of the lattice was calculated as the ratio of the applied load *P* to the solid cross-sectional area *BL*:

2 $$\begin{aligned} \sigma _{\textrm{rel}} = \frac{\sigma _{\textrm{lattice}}}{\sigma _{\textrm{solid}}}= \frac{P}{BL\sigma _{\textrm{solid}}}\,. \end{aligned}$$

The tensile strength of the PLA material was determined using standard specimens tested in accordance with the tensile test standard ASTM D3039^38^, yielding a mean value of 45 N/mm$^{2}$.

The total absorbed energy, $E_{\textrm{abs}}$, is defined as the area under the load-displacement curve, representing the integral of the compression load *P* over the end-shortening $\Delta$^39^:

3 $$\begin{aligned} E_{\textrm{abs}} = \int P\, \textrm{d}\Delta \,, \end{aligned}$$

where the integration limit for the $E_{\textrm{abs}}$ was determined based on the results of the pilot study presented in the “Results” section.

Following previous studies^40,41^, the specific energy absorption (SEA) per unit mass is calculated as:

4 $$\begin{aligned} \textrm{SEA} = \frac{E_{\textrm{abs}}}{m}\,, \end{aligned}$$

where $E_{\textrm{abs}}$ is the absorbed energy and *m* is the specimen mass.

An Ashby plot^21,42,43^ of relative strength versus relative density was used to evaluate the structural efficiency of the 3D-lattices. This approach enables direct comparison of different configurations and highlights how effective each design utilizes material to achieve strength.

## Results

This section presents the experimental results of the 3D-lattice structures under quasi-static compression. The analysis includes the evaluation of buckling behaviour through the measured load–displacement responses, covering ultimate load, post-buckling characteristics, and specific energy absorption. In addition, the deformation process is examined based on digital image correlation (DIC) results. Finally, the failure analysis is conducted to identify the dominant failure modes, as well as the defective components and their locations associated with each lattice configuration.

**Fig. 12:**
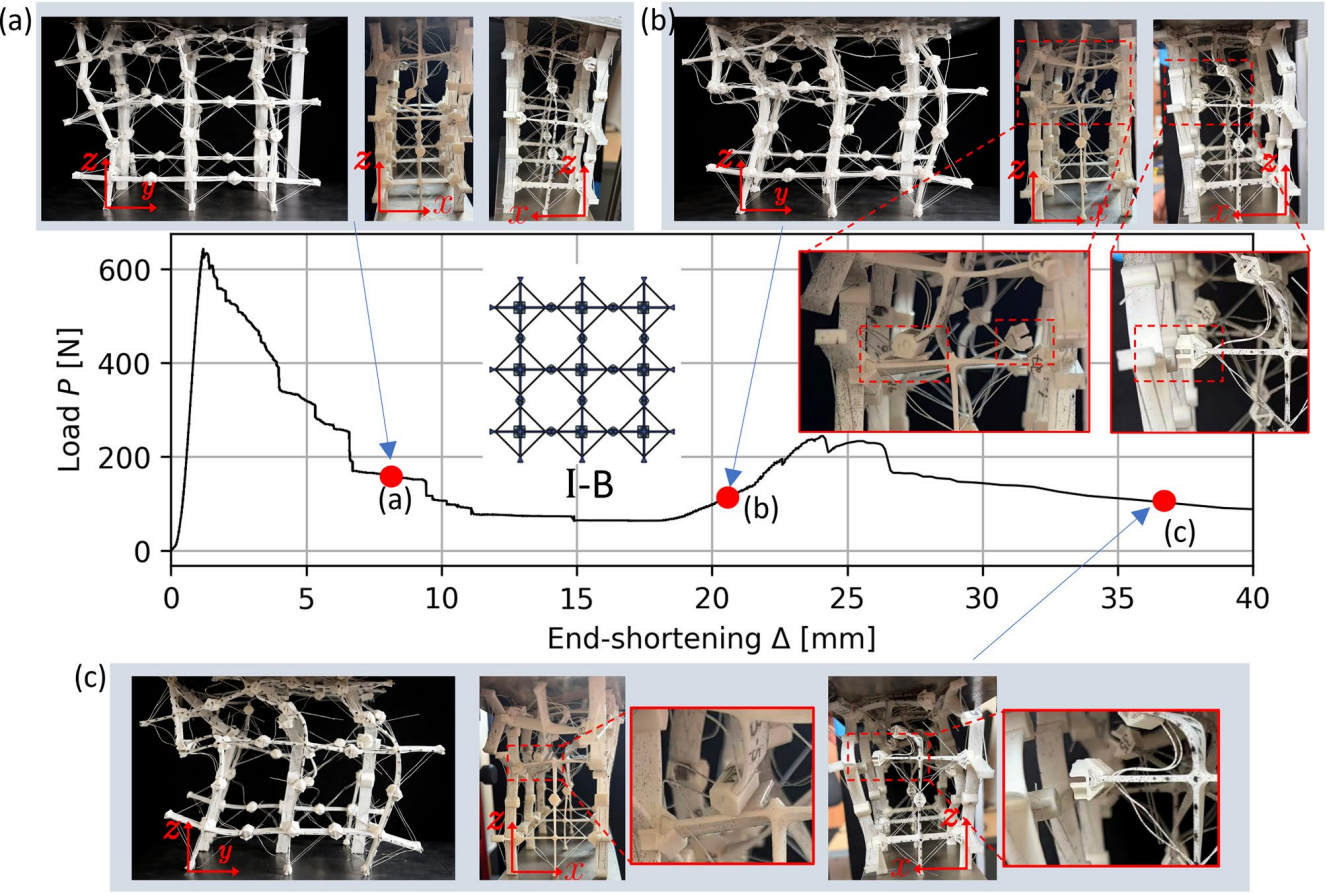
Deformation stages of the Type I-B 3D lattice (basic 3D lattice) under compression up to an end-shortening of 40 mm.

A compression test of Type I-B (basic 3D lattice) was conducted as a pilot study to identify an appropriate test range. This test provides the load–deformation response up to an end-shortening of 40 mm in Fig. 12. The structural behaviour in the initial elastic range and near the ultimate load, particularly the buckling response, is of primary importance in this study and is discussed in the following “Results” section. The behaviour after the peak load is the main focus of this pilot study, used to determine the test range based on deformation and failure progression. Based on this response, three distinct deformation stages can be identified:

- In the first stage, from the initial peak load to point (a) in Fig. 12, the stays in the unit cells (UCs) gradually detach, resulting in a progressive decrease in load. After this stage, plastic deformation of the UC columns can be observed, together with slight detachment of some connectors.
- In the second stage, around point (b) in Fig. 12, the beginning of densification can be observed, accompanied by an increase in load with further deformation. As shown in the corresponding deformed configuration, the upper part of the 3D lattice becomes compacted; void spaces are progressively closed, several structural members come into contact with each other, and some elements fracture. In addition, the different buckling deformations in the front and back $y-z$ planes induce rotation of the UCs in the $x-z$ plane, which further leads to detachment of the vertical connector (C-II).
- In the third stage, around point (c) in Fig. 12, the response again shows a relatively smooth decrease in load, which is associated with the progressive collapse and compaction of the second layer of UCs. More extensive densification is expected to continue until the remaining void space is fully closed. During the entire deformation process, no connectors or unit cells (UCs) were ejected from the structure or fell off, indicating stable deformation behaviour.

The design concept of the present study focuses on tuning the buckling behaviour of 3D stayed lattices. The deformation regime in which the stays actively influence the structural response, i.e. from initial buckling to early post-buckling, is of primary interest. Beyond this stage, the response becomes increasingly dominated by densification, characterised by extensive contact, compaction, and material crushing. In this regime, the influence of the stays is significantly reduced, and the behaviour is less sensitive to the designed geometric features. For this reason, the evaluation range was limited to an end-shortening of 7 mm, ensuring that the analysis focuses on the mechanically relevant deformation regime in which the structural design plays a dominant role.

### Buckling behaviour

The compression behaviour is compared across the groups identified in “Assembly” section, based on the different enhancement design methods.

#### Results of group I: basic stayed 3D-lattice

**Fig. 13:**
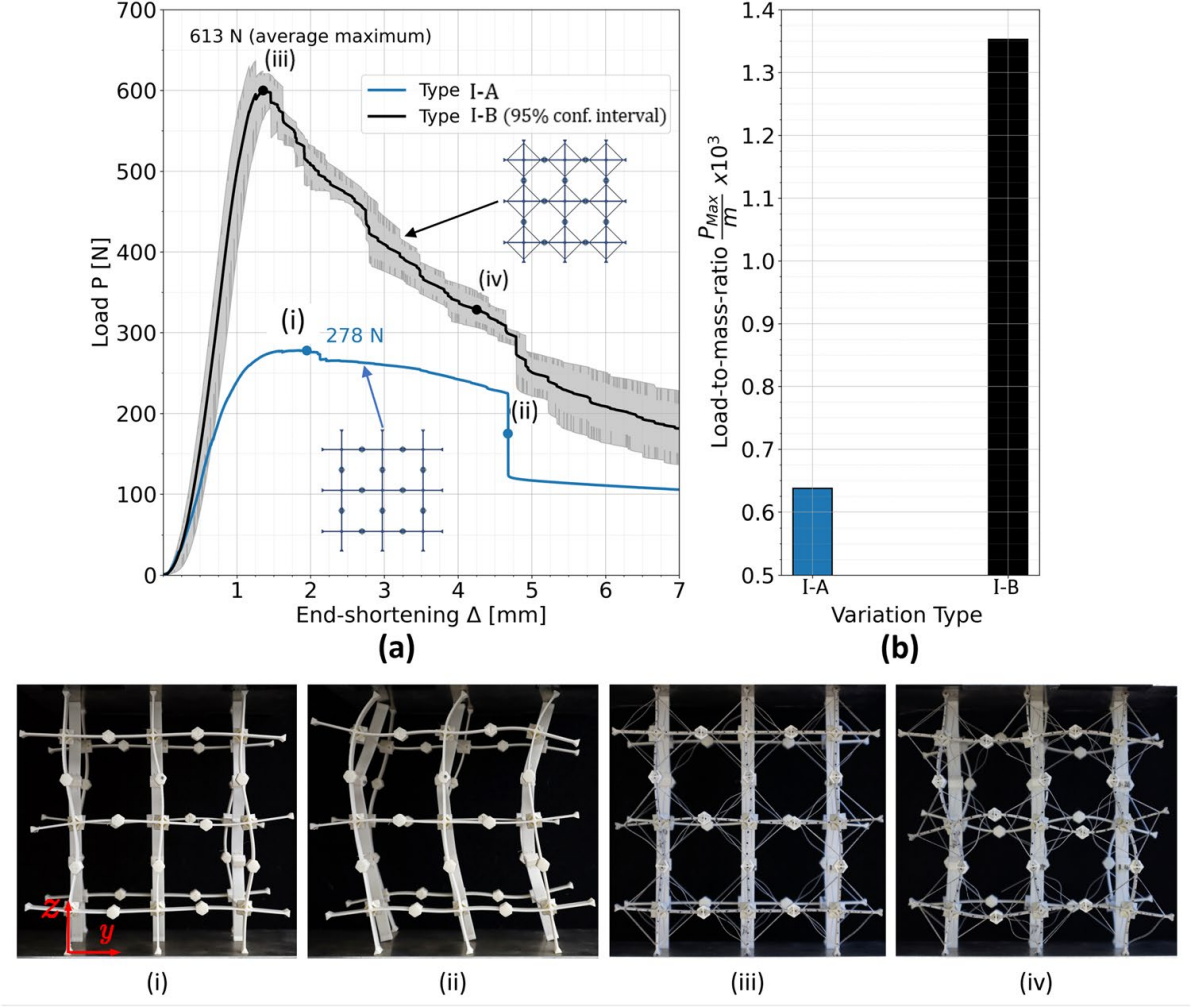
Compression behaviour of Group I basic stayed 3D lattices: (**a**) load (*P*) *vs.* end-shortening ($\Delta$) responses and (**b**) the corresponding load-to-mass ratios for different lattice types. Points (i–iv) in (**a**) indicate the corresponding deformed configurations at representative loading stages.

As shown in Fig. 13a, the relationships between the applied compression load (*P*) and the end-shortening ($\Delta$) for the 3D-lattice Type I-A (without stays, blue line) and Type I-B (with stays, black line) are presented. The stayed lattice (Type I-B) exhibited an ultimate load of 613 N, representing a 2.2-fold increase compared with the configuration without stays (Type I-A). Type I-B also exhibits a higher load-to-mass ratio, with only a slight increase in mass due to the addition of the stays. The lattice Type I-A exhibits a clear global C-mode buckling behaviour in the columns, while the cross-arms mainly undergo lateral displacement without significant deformation, as shown in Fig. 13(ii). This deformation mode corresponds to a lower critical load, consistent with Euler’s buckling theory.

In contrast, the Type I-B lattice, with the introduction of stays, exhibits a higher-order S-mode buckling pattern, as shown in Fig. 13(iii), which is associated with a higher critical load. This can be attributed to the lateral restraint provided by the stays, which promotes a transition from a global C-mode to an S-mode buckling behaviour. Furthermore, due to the presence of the stays, the cross-arms are also subjected to compressive forces and actively participate in the buckling process. This interaction leads to the formation of a double S-mode deformation at the unit cell level, thereby enhancing the overall load-bearing capacity of the structure.

During the post-buckling phase of the Type I-B lattice, the applied load decreases with increasing end-shortening. Progressive detachment of the stays occurs at various locations, leading to a further reduction in load capacity. As more stays become detached, the buckling mode evolves from an initial S-mode to a global C-mode configuration, as shown in Fig. 13(iv).

Through the comparison of the ultimate load and post-buckling behaviour, it is evident that the stay effect can be transferred from the 2D-lattice (approximately a 4.0-fold increase in load capacity^19^) to the 3D-lattice structure (about a 2.2-fold increase). The narrow shaded region surrounding the black curve denotes the 95% confidence interval for Type I-B, demonstrating strong repeatability and the reliability of the measured data. This confirms that the manufacturing process of the 3D-lattice is consistent and reliable, achieved through the assembly of unit cells using different types of connectors.

#### Results of group II: 3D-lattices with additional components

**Fig. 14:**
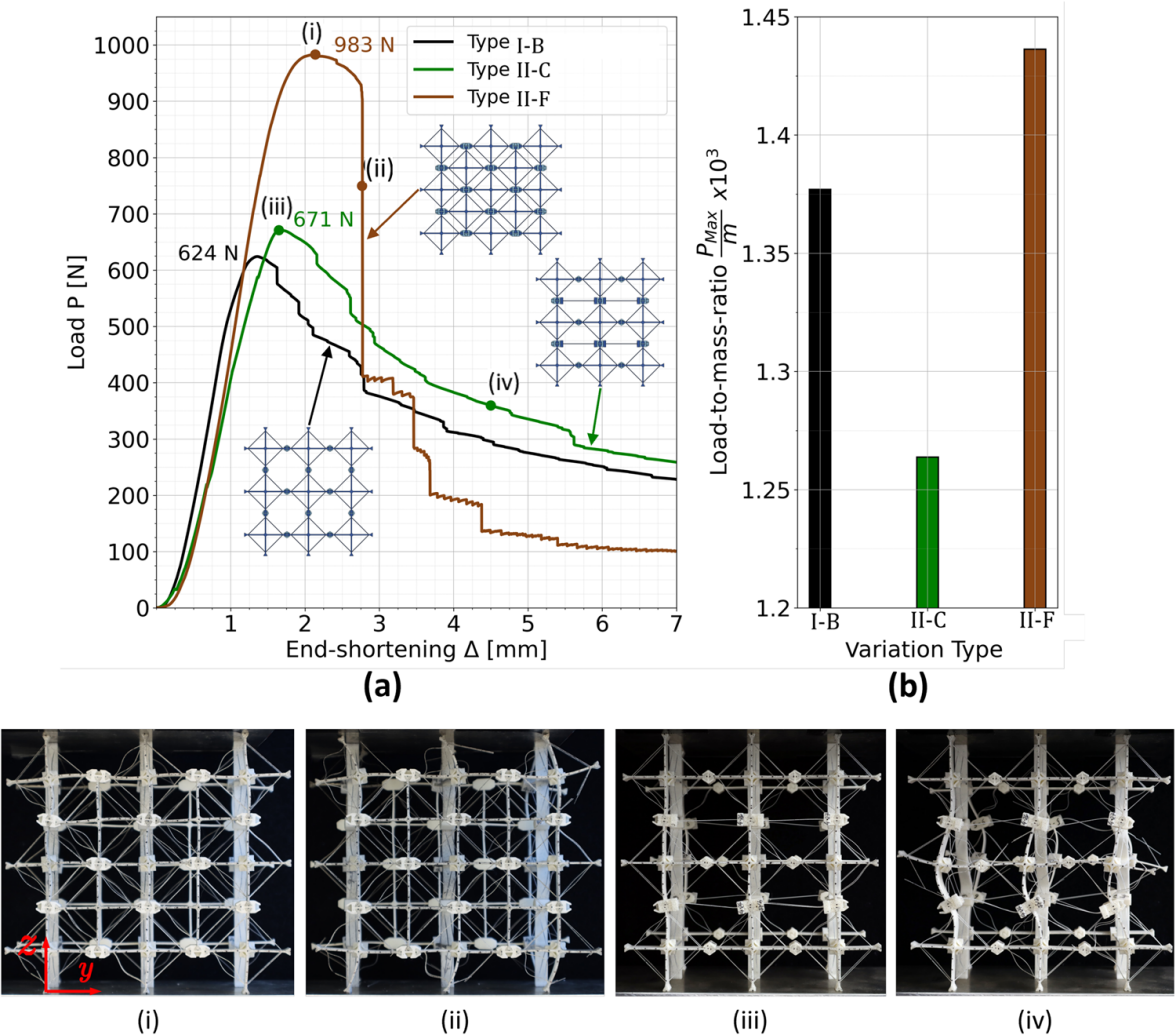
Compression behaviour of group II 3D-lattices with additional components: (**a**) load (*P*) *vs.* end-shortening ($\Delta$) responses and (**b**) the corresponding load-to-mass ratios for different lattice types. Points (i–iv) in (**a**) indicate the corresponding deformed configurations at representative loading stages.

Building on the basic 3D-lattice concept, an approach involving the addition of extra structural components to further enhance the load-bearing capacity was applied. The results, shown in Fig. 14, compare Type II-C (green line), which includes additional bracings, and Type II-F (brown line), which incorporates extra basic unit cells.

With the aid of the bracings, the ultimate load of Type II-C increased slightly to 671 N. However, the additional bracings resulted in a noticeable increase in mass, leading to a slight reduction of about 7.8% in the load-to-mass ratio relative to the basic stayed lattice (Type I-B). Although the added bracings do not provide a positive effect in terms of lightweight design requirements, they influence the buckling behaviour of the lattice structure. Without bracings, the columns of the lattice tend to buckle in different directions, leading to large deformations as neighboring columns deform inconsistently. The incorporation of bracings aligns the buckling direction of adjacent columns, promoting a more uniform and controlled deformation mode across the lattice.

The bracings function only as stays, providing tensile restraint rather than compressive support. In contrast, the introduction of additional basic unit cells enhances the structural performance more effectively. The Type II-F lattice demonstrated a 57% increase in ultimate load compared with Type I-B, accompanied by a 4.3% rise in total mass. However, after reaching the ultimate load, the structure experienced a sudden and significant drop in load capacity due to the simultaneous failure of multiple stays, as shown in Fig. 14(ii). The sudden drop in load-bearing capacity is unfavourable, as it reflects a brittle failure mode and limited energy absorption capability.

#### Results of group III: 3D-lattices with different unit cells

**Fig. 15:**
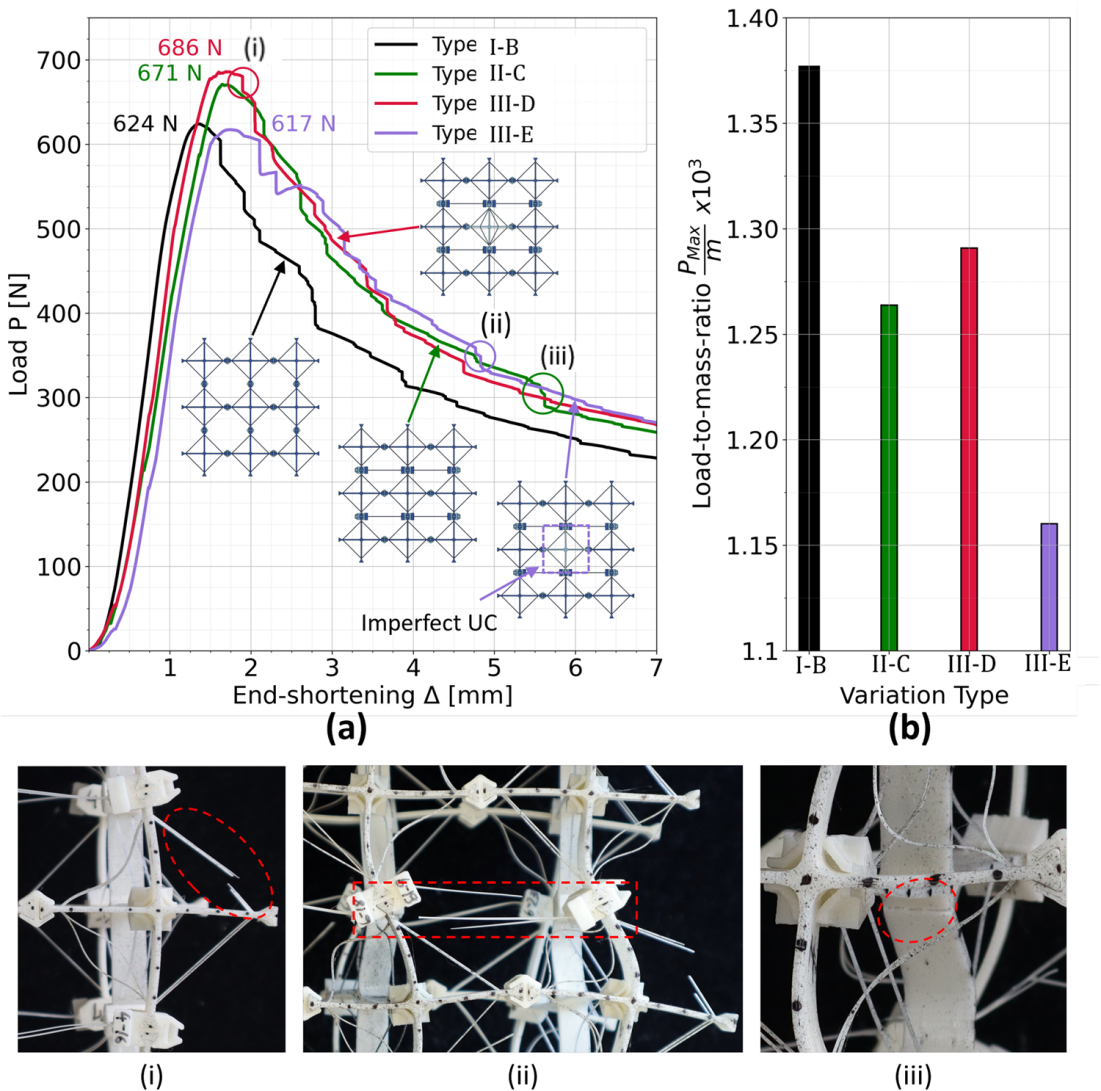
Compression behaviour of group III 3D-lattices with different unit cells: (**a**) load (*P*) *vs.* end-shortening ($\Delta$) responses and (**b**) the corresponding load-to-mass ratios for different lattice types. Points (i–iii) in (**a**) indicate the corresponding deformed configurations at representative loading stages: (i) stay detachment in the UCs, (ii) breakage of stays in the bracing elements and (iii) column failure in the UCs.

In this group (see Fig. 15a), the central unit cells were assigned with different specialized UCs to tune the buckling behaviour, particularly at the local level. The lattice Type III-D (red line) incorporates enhanced unit cells at the centre, and this form of enhancement is reflected in a slight increase in the ultimate load compared with Type II-C. During the post-buckling stage, the behaviour is consistent with most other cases, with the load gradually decreasing as the stays detach.

The Type III-E lattice exhibits a different tendency, with the ultimate load decreasing by 8.7% compared with Type II-C. The introduced imperfect unit cell has been shown to have a positive effect on the ultimate load and can be treated as an enhanced unit cell at the centre. However, when it is integrated into a 3D-lattice, it leads to a reduction in load capacity. This indicates that 3D-lattices are sensitive to geometric imperfections.

These two lattice configurations demonstrate that the buckling behaviour can be effectively tuned by placing different types of unit cells at specific locations within the structure, thereby enabling the achievement of different design objectives such as enhanced load capacity or tailored post-buckling responses.

#### DIC results

**Fig. 16:**
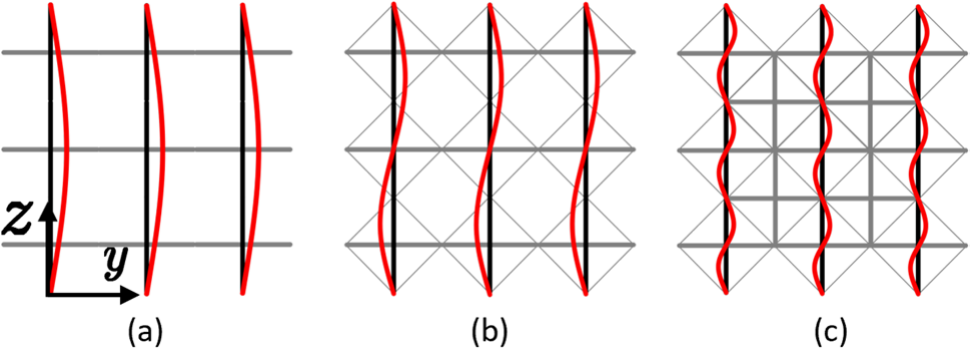
Theoretically possible buckling modes of the 3D-lattice in the $y-z$ plane: (**a**) global C-mode, (**b**) global S-mode and (**c**) local S-mode.

The theoretically possible buckling modes of the 3D-lattice in the $y-z$ plane are illustrated in Fig. 16. Without the restraint of the stays, as shown in Fig. 16a, the column exhibits a global C-mode buckling pattern. With partial restraint from the stays, the column may buckle in a global S-mode, as depicted in Fig.  16b. When the restraint is sufficiently strong, the column can develop a local S-mode buckling pattern, as shown in Fig. 16c. The corresponding buckling load increases significantly with the degree of restraint.

The global C-mode is clearly observed in the Type I-A structure without stays (see Fig. 13b), where all columns buckle in the same direction. However, the buckling mode of the structure with stays is neither a distinct global S-mode nor a local S-mode. Instead, it exhibits a more complex, mixed behaviour of both modes. The transition from a global C-mode to a mixed S-mode is attributed to the introduction of the stays, which provide additional constraints to the system, leading to an increased buckling load.

The initial buckling of the structure occurs within a very short time and at very small deformation levels, which are not directly observable under conventional visual inspection. In this context, DIC proves to be a valuable tool for capturing the onset of buckling and identifying the corresponding deformation modes. With increasing end-shortening, the deformation amplitude becomes sufficiently large to be visually observed, especially when stay failure occurs, leading to a transition from S-mode back to C-mode behaviour. Hence, the DIC data analysis is focused around the ultimate load to capture the buckling behaviour.

**Fig. 17:**
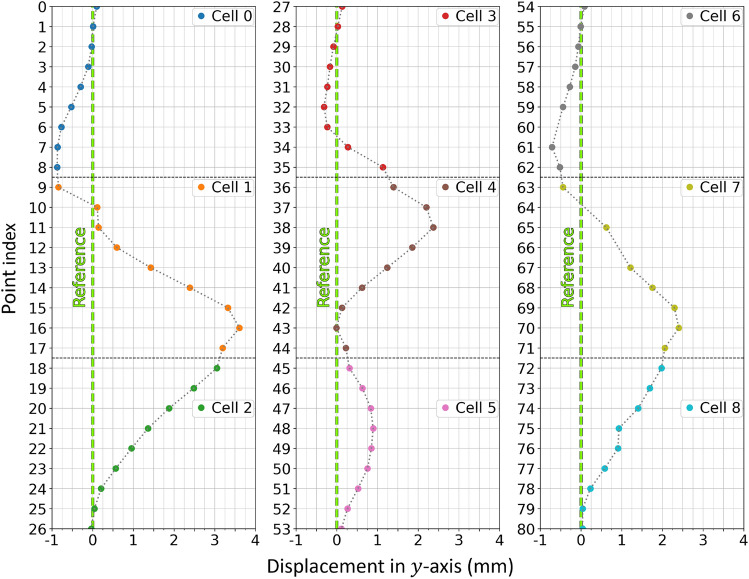
DIC results of the Type I-B lattice illustrating the deformation pattern and buckling mode in the $y-z$ plane at the maximum load.

Figure 17 illustrates the real deformation of Type I-B structure in the $y-z$ plane. Combined with DIC results, it precisely displays millimeter-level deformations. The dots in the figure indicate the positions of tracking points on the column, while the polylines represent the simulated deformation shape of the column. This deformation state corresponds to the structural condition under maximum loading.

Each column exhibits its own deformation pattern. The middle column tends more toward local s-mode buckling, while the columns on both sides show characteristics of a global S-mode buckling. Only the unit cell at the center displays a distinct local S-mode, whereas the other unit cells near the boundaries are influenced by boundary conditions and has different deformations. The connectors also undergo a certain degree of lateral displacement, causing each unit cell to experience not only buckling but also local movement and rotation. The global buckling directions of the three columns are not perfectly aligned.

**Fig. 18:**
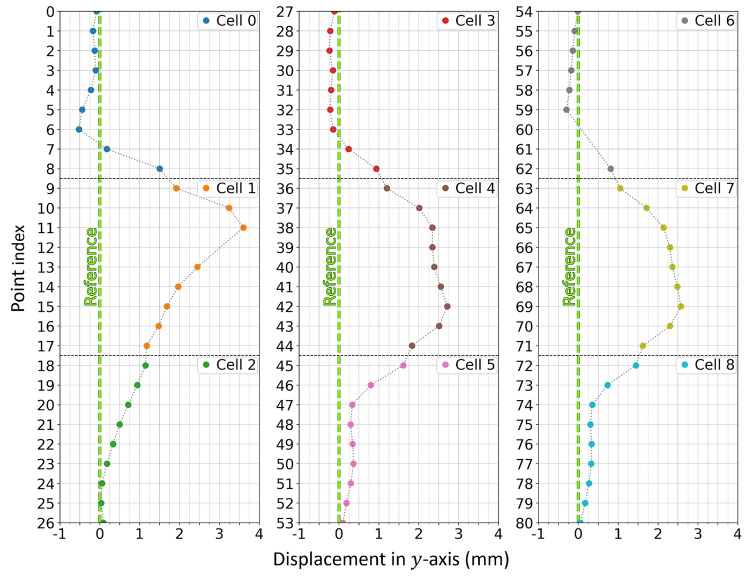
DIC results of the Type III-D lattice illustrating the deformation pattern and buckling mode in the $y-z$ plane at the maximum load.

The DIC analysis of the Type III-D structure (with enhanced unit cells) shows a different buckling mode, characterized by greater deformation in the middle layer (cells 1, 4, and 7). The effect of the bracing is visible, as it helps align the buckling direction of all columns. The locally applied enhanced unit cells influence the overall buckling mode of the entire structure. The connectors in the Type III-D exhibit larger displacements than those in the Type I-B (Fig. 18).

In general, DIC can be effectively used to capture the deformation of this type of structure. However, it is difficult to identify the pure buckling behaviour of each unit cell, as it is also influenced by the movement of the connector locations. The S-mode observed in the central unit cells suggests that improving overall performance could be achieved by increasing the lattice density to incorporate more inner unit cells.

### Specific energy absorption

**Fig. 19:**
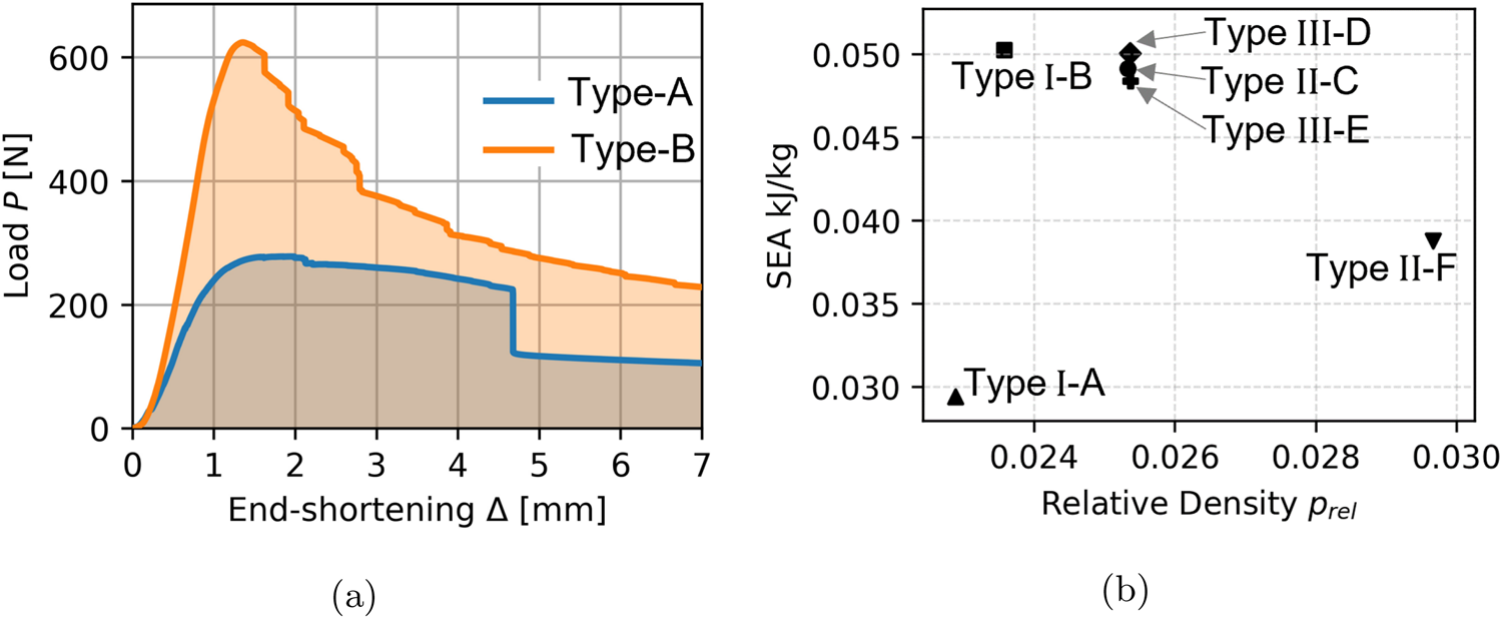
Results of energy absorption: (**a**) total energy absorption of lattice Types A and B and (**b**) comparison of different lattice configurations in terms of specific energy absorption (SEA) versus relative density ($\rho _{\textrm{rel}}$).

Figure 19a illustrates the energy absorption behaviour of lattice Types A and B, represented by the area under their respective load–end-shortening (*P*–$\Delta$) curves. The energy absorption of Type I-B (2.39 J) is approximately 81% higher than that of Type I-A (1.32 J), demonstrating the significant contribution of the stays to the overall energy absorption capacity.

Figure 19b illustrates the relationship between specific energy absorption (SEA) and relative density ($\rho _{\textrm{rel}}$) for all lattice types. Type I-B achieves the highest SEA at a relatively low density, indicating excellent energy absorption efficiency, while Type II-F, despite its higher density, shows reduced SEA due to its increased mass. The weak energy absorption behaviour of Type II-F is attributed to the sudden load drop that occurs immediately after reaching the maximum load (see Fig. 14). Types C and D exhibit improved energy absorption compared with the basic stayed lattice (Type I-B), owing to the additional bracings and enhanced unit cells that increase the ultimate load. Type III-E, however, shows slightly lower energy absorption with its imperfect unit cells. The energy absorption trend of the three lattice types corresponds closely to their ultimate load behaviour.

The SEA for Type I-B (basic 3D lattice) based on the data shown in Fig. 12, evaluated up to 40 mm, reaches 6.69 J , which is substantially higher than the value obtained at 7 mm (2.39 J). This demonstrates the more complete energy absorption capacity of the structure when densification is fully considered.

### Failure analysis

**Fig. 20:**
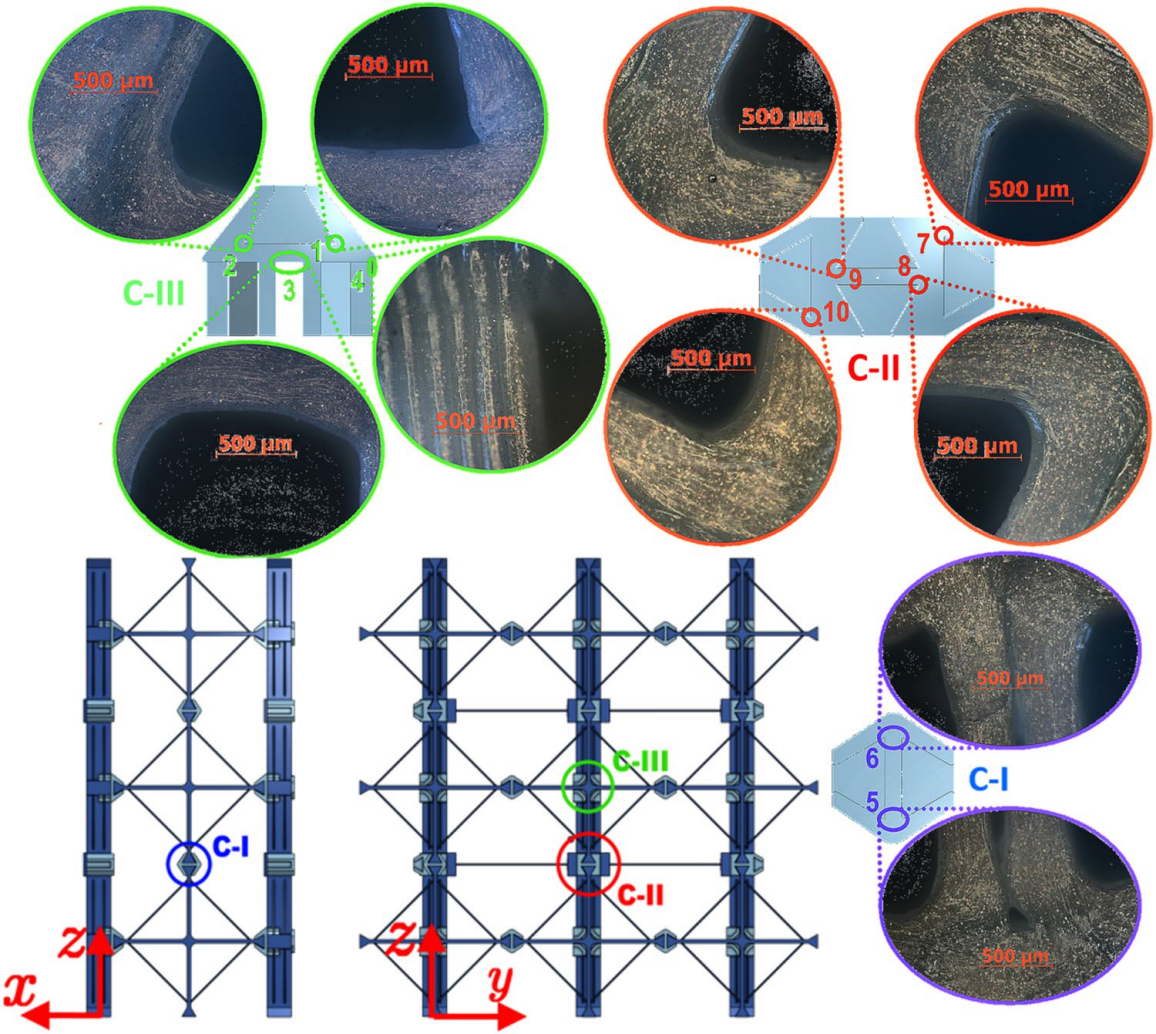
Microscopic examination at 50 $\times$ magnification of the three connector types after loading at different locations.

In the post-buckling phase, as the end-shortening increased, the load gradually decreased. However, due to the large deformations, some components within the 3D-lattice structure experienced local failures, leading to a reduction in the overall load-bearing capacity. Each component of the structure was investigated:

- *UCs*
  As shown in the Fig. 15i, were broken due to the large tensile stresses induced by the significant deformations. The failure of these stays occurred progressively and randomly in different UCs. As more stays failed, the overall load-bearing capacity decreased correspondingly. There is no sharp reduction in the post-buckling load-displacement response, because once one of the stays fails, the internal stresses are redistributed among the remaining load-bearing members. This redistribution mechanism allows the structure to sustain its overall stability and maintain load transfer capability. The progressive failure of additional stays leads to a gradual degradation of the structural stiffness and load-bearing capacity, rather than an abrupt collapse. However, Type II-F is an exception. After reaching the ultimate load, a sudden failure occurs in the early post-buckling stage, characterized by a sharp load drop. This behaviour is because of the simultaneous failure of multiple stays within several unit cells, resulting in a rapid loss of structural integrity (see Fig. 14).
  As shown in Fig. 15iii, when the global deformation becomes sufficiently large (in Type II-C, with approximately 5.5 mm end-shortening), the columns within the unit cells exhibit plastic deformation. In Type II-C, local cracking can be observed in the columns, indicating the onset of material failure. However, this phenomenon is rare and does not represent the structure’s general behaviour.
- *Bracing*
  The additional reinforcement component (bracing), can also fail under large end-shortening, as illustrated in Fig. 15ii. When the spacing between two columns increases due to local buckling, the stays within the bracing are subjected to excessive tensile stress and may fracture accordingly.
- *Connector*
  Although the most obvious failures were primarily caused by the breakage of the stays, the connectors play a crucial role in this design concept and therefore deserve careful examination. No significant cracking or plastic deformation of the connectors was observed during visual inspection. All connectors remained securely in their designated positions throughout the entire testing process up to an end-shortening of 7 mm, with no instances of popping out.
  Furthermore, each connector type was examined using an microscope with 50 $\times$ magnification to identify potential micro-cracks or defects, particularly in critical regions such as corners and junctions. The microscopic observations are presented in Fig. 20. No visible damage was detected, indicating the high quality of the connectors and providing strong support for the modular assembly concept. The absence of any observable defects suggests that the connectors can be reused multiple times.
  However, with increasing end-shortening up to 40 mm, including the densification phase, connector detachment is observed. A notable phenomenon occurs in Type I-B, where asymmetric buckling in the front and back $y-z$ planes induces rotation in the $x-z$ plane, leading to significant detachment of the vertical connector (C-II) in Fig. 12c.

## Discussion

The discussion of this work divided into two parts: the first part focuses on the 3D-lattice assembly design and the MEX additive manufacturing process, while the second part analyses the buckling behaviour of the structure under compression experiments. After this, the performance of the developed 3D-lattice structure is compared with previous studies to highlight its achievements, potential applications, and possible improvements.

### Discussion of the 3D-lattice structure design

The successful transition from a 2D to a 3D-lattice configuration was realized using a modular assembly concept, in which various connector types were employed to construct unit cells into a 3D framework. This accomplishment achieves the first research objective of the present study.

#### Unit cells

The triangular redesign of the unit cell tips adopts the mortise–tenon concept with dovetail-shaped tips, forming complementary male–female interfaces that interlock precisely.

Besides the improvements made to the CAD model, the printing parameters for the bridging (stays) were adjusted independently from the overall printing settings. This approach allowed a lower printing speed for the stays while increasing the speed for other parts, enabling more accurate control and potential time savings. This adjustment is particularly beneficial for 3D-lattices, which require the mass production of numerous unit cells.

#### Connectors

The various connectors are designed to accommodate multiple connection scenarios, enabling not only the assembly of unit cells but also the integration of additional enhancement components, such as bracings. This provides a high degree of design flexibility and adaptability. The connectors exhibited excellent performance under varying compression loads. Microscopic examination revealed no cracking, and all connector joints remained intact even during large structural deformations, demonstrating stable mechanical integrity across different connector types and locations.

Although the connectors realize the modular assembly and offer significant advantages, their mass proportion within the 3D-lattice is relatively high, ranging from approximately 28.37% to 35.04% (see Table 3), which is not ideal for achieving a lightweight structure. Future improvements could focus on reducing the connector weight through design optimization. Since no cracks were observed in the connectors after loading, there remains potential to slightly lower their load-bearing capacity to save material. Another approach would be to decrease the number of connectors by printing certain components as integrated parts. This represents a trade-off between assembly flexibility and material efficiency.

The present study is limited to PLA as the base material, as PLA is one of the most widely used materials in MEX-AM and therefore provides a representative basis for the investigation. Future work should consider a wider range of materials tailored to specific applications. In particular, extending the stayed lattice concept to more ductile materials represents a promising direction for enhancing energy absorption performance. Furthermore, the use of higher-strength materials may improve the load-bearing capacity of the structure and reduce premature failure in critical components, such as the stays.

#### Modular assembly concept

The modular assembly concept offers a high degree of design freedom after manufacturing. This approach enables the creation of more complex geometries compared with the simple cubic structure presented in this work. It has the potential to support structural design modifications according to specific application requirements after additive manufacturing. Moreover, since the connectors showed no visible micro cracks after loading, they may also be reused in subsequent assemblies, enhancing the sustainability of the process.

The modular assembly concept was developed to address the limitation of conventional MEX printers, which can only perform planar printing. As a result, the stays can currently be printed only within the layer plane rather than in out-of-plane directions. With the advancement of manufacturing technologies, non-planar printing may become feasible, allowing the complete 3D-lattice to be fabricated in a single process, with the stays printed freely in space. This would significantly enhance the structural performance, enabling reduced mass while maintaining or even improving load-bearing capacity.

For example, robotic additive manufacturing (RAM) systems used in civil engineering applications can perform spatial printing beyond the planar constraint^44–46^. Other technologies, such as the printing systems with rotating build platforms, also demonstrate the potential for multi-directional fabrication^47,48^. In the case of polymer extrusion, it may be possible to redesign the extruder with a rotational axis and employ fully customized G-code control to achieve non-planar 3D printing^49,50^. However, these techniques are still in an early stage of development and not yet easily applicable to general use. Therefore, the modular assembly concept remains a practical and effective solution for current manufacturing conditions.

### Discussion of the buckling behaviour

Lattice structures with six different configurations were tested under uniaxial compression to examine their buckling and post-buckling behaviour, addressing the second objective of this study.

#### Stay-enhancement concept in 3D-lattice

In general, the stay-enhancement concept was successfully introduced from the 2D lattice to the 3D-lattice. The lattice with stays (Type I-B) exhibited a 2.2-fold improvement in load-bearing capacity compared with the lattice without stays (Type I-A), while requiring only a minor increase in material usage (3.1%) for the stays. The addition of stays alters the buckling mode from a global C-mode to a higher-order buckling mode, which is consistent with the expected behaviour.

However, this enhancement is lower than the load-increasing factor observed in the 2D lattice, which reached approximately 4.4^19^. The possible reason for this difference is related to the buckling behaviour. With the introduction of stays in the unit cells, the overall buckling mode is no longer a purely global C-mode. However, it is also not a completely local S-mode within individual unit cells, due to the limited restraint at the connection regions between adjacent UCs. The actual buckling response represents a combination of both global and local modes (based on the DIC results), resulting in a mixed buckling pattern, which leads to a critical load that falls between the values predicted for the global and local buckling modes. In order to achieve a local S-mode, stronger constraints are required within the system, for example through the introduction of lateral restraint to the connectors. This observation inspired the design of Group II: 3D-lattices with additional components.

In addition, when considering the density of the lattice, the 3D-lattice does not exhibit the same level of efficiency as the 2D-lattice. This is primarily due to the additional connector mass, which mainly serves a joining function but contributes little to load-bearing capacity, thereby reducing the overall structural efficiency. With further advances in additive manufacturing techniques, enabling the fabrication of 3D lattices in a single process without discrete connectors, the structural efficiency of 3D lattices is expected to be significantly improved.

#### 3D-lattices with additional components

To improve the restraint at the connection, additional bracing (in the Type II-C) and UCs (in the Type II-F) are introduced, increasing the ultimate load compared to the structure without stays, with values of 2.41 and 3.53 respectively.

To address this issue of asymmetric buckling in $y-z$ planes and vertical connector (C-II) detachment, Type II-C with additional bracing elements have been developed. The DIC results show that these bracing components can effectively align the buckling directions, thereby reducing connector detachment. As a result, the structure can sustain a more stable and extended post-buckling deformation path, which is beneficial for energy absorption performance. Meanwhile, as the bracing provides only tensile restraint, it results in only a slight improvement in the ultimate load.

The additional UCs are significantly more effective and provide much higher restraint in both tension and compression in Type II-F. Consequently, lateral movement of the connection is reduced and it is more prone to local S-mode buckling. However, the additional UCs in Type II-F lead to a significant mass increase of 13% compared to Type I-B. High stress in the structure means that more stays break at the same time after the peak load, leading to sudden failure, which is not ideal for real-world applications.

#### 3D-lattices with different unit cells

Two different types of unit cells (UCs) were introduced in the middle region of the 3D-lattice to modify the local behaviour and tune the overall buckling response. Type III-D, incorporating enhanced UCs, exhibited a slightly positive influence on structural stability. This confirms that introducing different UC configurations locally is an effective strategy for tuning the global buckling behaviour. However,Type III-E with imperfect UCs, had a negative effect on the overall performance. This trend differs from the results of the single-UC study^20^, suggesting that imperfections in the 3D-lattice are more sensitive to interactions between adjacent cells than to the behaviour of an isolated unit cell.

#### General summary and comparison with previous work

**Fig. 21:**
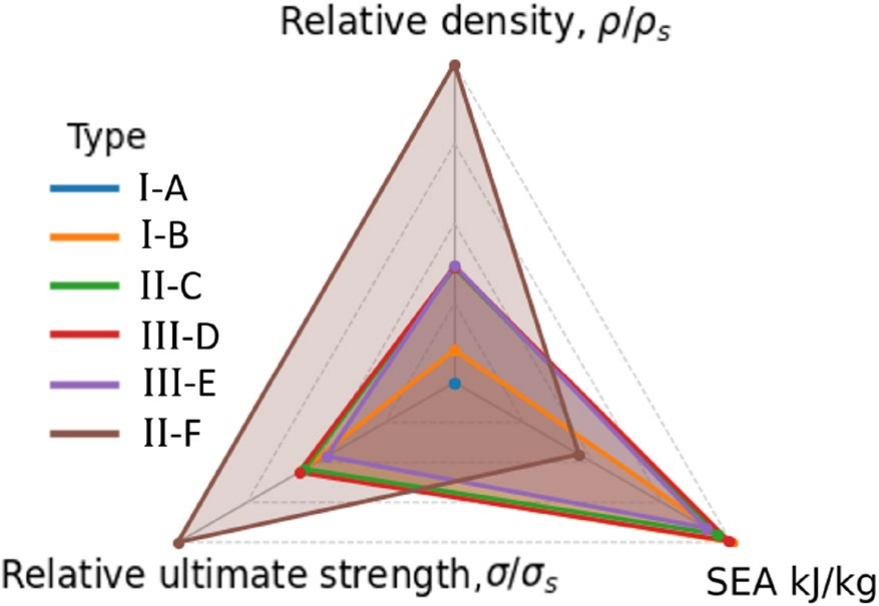
Comparison of lattice types in terms of relative density, relative ultimate strength, and specific energy absorption (SEA).

Figure 21 illustrates an overall comparison of the six lattice types in terms of relative density, relative ultimate strength, and specific energy absorption (SEA). Depending on the intended application, the most suitable lattice type can be selected accordingly. For applications requiring higher load-bearing capacity, Type II-F shows strong potential, offering a good balance between strength and density. In contrast, if structural stability under large deformation or enhanced energy absorption capacity is prioritized, Types III-D demonstrate better performance.

**Fig. 22:**
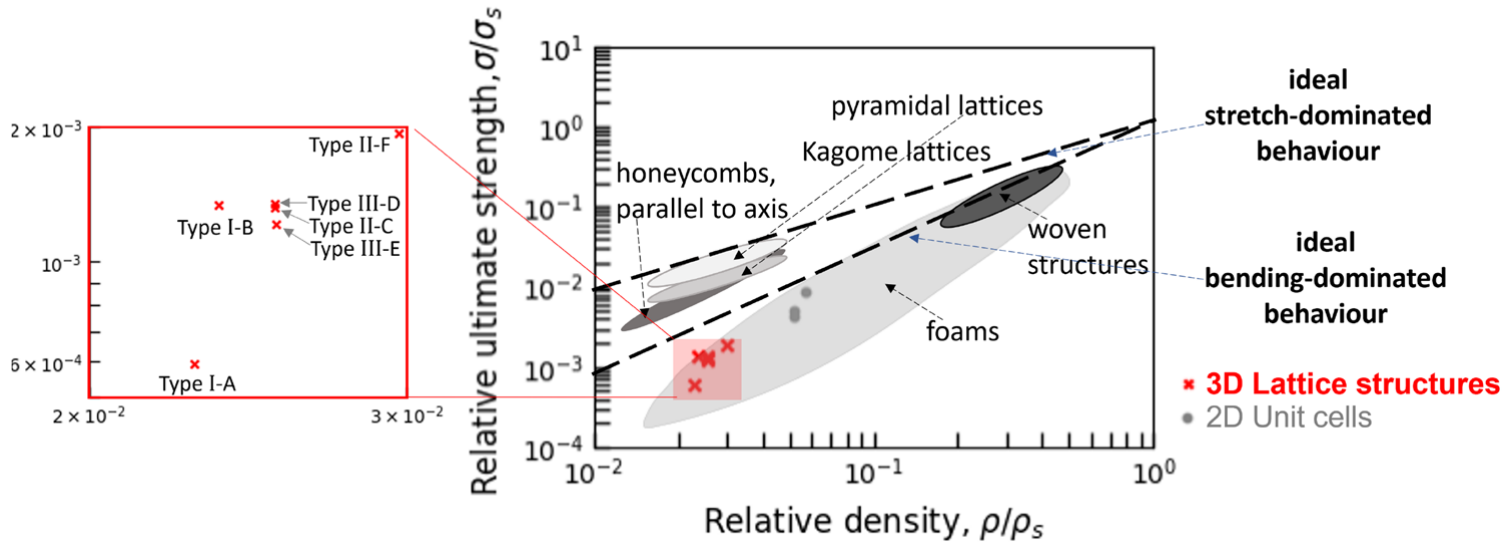
Ashby chart^43^ of relative strength ($\sigma / \sigma _s$) versus relative density ($\rho / \rho _s$) for various cellular and lattice structures, with the present work highlighted with red cross markers.

In the Fig. 22, the results of current work are highlighted with red cross marker in the Ashby chart^21,43^ to illustrate the relationship between relative strength ($\sigma / \sigma _s$) versus relative density ($\rho / \rho _s$). The relative strength of the 3D-lattices remains largely within the range expected for bending-dominated structures. Their data points are located in the region typically occupied by foams. The enlarged plot shows that the order of relative strength closely follows that of the specific energy absorption (SEA) (see Fig. 19b), except for Type II-F, which exhibits the highest relative strength but comparatively lower energy absorption capacity.

#### Limitation and future work

Regarding the mechanical testing conditions, the present study is limited to quasi-static uniaxial compression at a low loading rate, which is suitable for observing the buckling behaviour in detail. However, for broader engineering applications, more complex loading scenarios and boundary conditions should be considered. In particular, dynamic loading conditions are highly relevant for energy absorption performance and should be investigated in future work. For DIC, a more detailed quantitative analysis of the full-field deformation (e.g., local displacement, curvature, and stress distribution) would provide further insight into the underlying mechanisms and is considered a promising direction for future work.

In the pilot study (up to an end-shortening of 40 mm in Fig. 12), the relatively long test duration resulted from the low loading rate of 0.2 mm/min. For future investigations focusing on densification and energy absorption, the test procedure could be further optimised to reduce the total testing time. To determine the suitable speed, a series of parametric studies is required, as PLA exhibits strain-rate-dependent behaviour^51–53^, which may significantly influence the mechanical response and energy absorption performance.

In addition, previous studies have employed elastic stability theory^54^ to simulate the non-linear buckling behaviour of unit cells (UCs), using reduced degrees of freedom. Early analytical models adopt discrete representations based on rigid links and linear springs, which enable the prediction of the fundamental instability modes, namely the global C-mode and the double S-mode^55–57^. More recent studies have introduced nonlinear spring models to capture the mechanical response of the stays, enabling the simulation of their influence on the structural behaviour, including the occurrence of secondary bifurcation^58^. These approaches provide reliable predictions at the UC level.

However, extending these models from 2D unit cells to full 3D-lattice structures remains challenging, owing to the increased geometric complexity and the coupling between local and global deformation modes. Accurately capturing the critical points associated with bifurcation further complicates these challenges. In the future work, numerical simulations (such as FEM)^59^ and AI-driven data approaches^60^ may provide improved capabilities to predict and optimise the behaviour of more complex lattice structures.

To further improve the buckling behaviour of the structure and better meet the design requirements of lightweight structure, beyond the mass-reduction strategies discussed in the previous “Discussion of the 3D-lattice structure design” section, several additional aspects could be explored with respect to buckling behaviour. Optimizing the arrangement of unit cells and reducing their individual size may significantly increase the ultimate load capacity^20^. And employing more enhanced unit cells^34^ to construct the entire 3D-lattice could lead to higher-mode buckling and improved load-bearing capacity, with only a minor increase in material usage.

#### Potential applications

This stayed 3D-lattice structure demonstrates significant advantages in mechanical properties, holding great potential for lightweight and energy-absorption applications. Its manufacturing process can efficiently utilize additive manufacturing technology, achievable without requiring expensive or complex equipment. It is particularly appropriate for use in the automotive and aerospace industries, where structures that are lightweight yet have a high energy absorption capacity are required^4,5,61,62^.

## Conclusion

This work has successfully introduced a modular design concept for stayed 3D-lattice structures, which allows the stays to be arranged in non-planar directions.

*Additive manufacturing and modular design*

- The printing parameter settings were optimised, enabling separate tuning of the printing speed for the main lattice and the stays (bridging).
- Tolerances at the connection points were well controlled, ensuring reliable assembly quality.
- Three different connector types provide high design flexibility, enabling the future development of more complex and stronger stayed 3D-lattice structures. The four-way connector (Type C-III) exhibits the highest potential because it enables connections in different orientations as well as between distinct components.
- Modular design facilitates disassembly, thereby enhancing recyclability and supporting a design-for-recycling strategy.

*Buckling behaviour and energy absorption*

- Uniaxial compression tests confirmed that the stay concept can be effectively applied in three dimensions, increasing the ultimate load by up to a factor of 3.53.
- The energy absorption capacity was significantly enhanced by the stays, reaching approximately 81%.
- The buckling behaviour could be tuned across five lattice configurations by varying unit cells and reinforcement strategies.
- Failure at large deformations (up to 7 mm) mainly occurred through detachment of the stays, while the connectors remained intact without observable micro-cracks.
- Digital Image Correlation (DIC) enabled detailed observation of the buckling modes and local deformation behaviour.

This work provides different 3D-lattice designs and analyses including relative density, relative strength, and specific energy absorption, allowing the selection of the most suitable modular design concept based on the intended application.

## Data Availability

Raw data from theexperiments can be provided upon request from the corresponding author. Data for additive manufacturing of the 3D-lattice Cell presented in this work are provided on GitHub in a subfolder of the repository: https://github.com/SVFS-TUBerlin/Publications_Supplementary_Materials/tree/main/2026_OU_Scientific%20Reports_3D_stayed_lattice.
